# Supplementary material for: Practical AI-based cell extraction and spatial statistics for large 3D bone marrow tissue images
Source: Cell Rep Methods. 2026 Mar 13;6(3):101334. doi: 10.1016/j.crmeth.2026.101334 (PMC13030987; doi:10.1016/j.crmeth.2026.101334)
Supplement: Document S1. Figures S1–S4 [file mmc1.pdf]

Cell Reports Methods, Volume *6*

Supplemental information

Practical AI-based cell extraction and spatial statistics for large 3D bone marrow tissue images

George Adams, Floriane S. Tissot, Chang Liu, Cera Mai, Chris Brunsdon, Ken R. Duffy, and Cristina Lo Celso

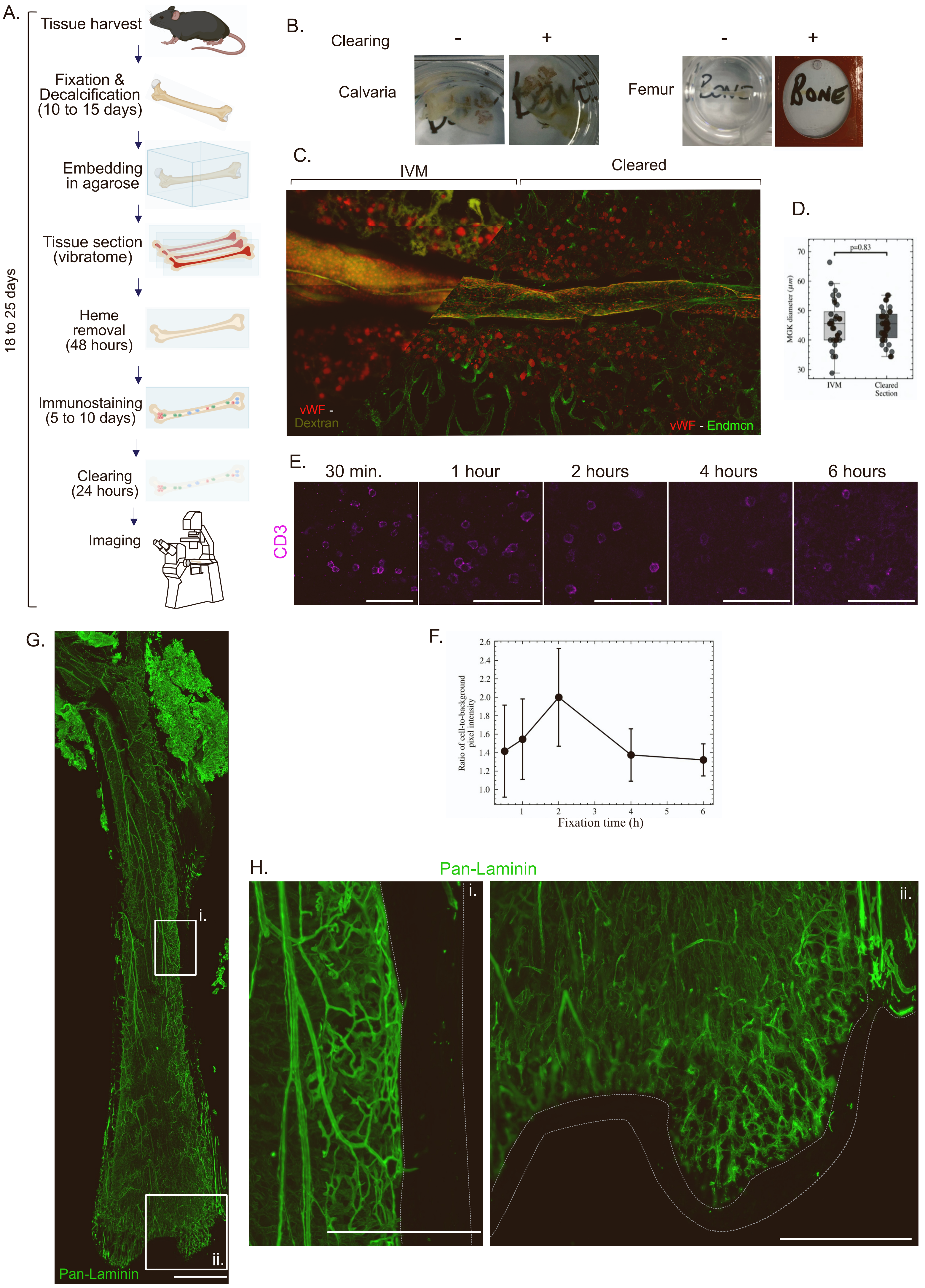
A.
Tissue harvest
Fixation & Decalcification (10 to 15 days)
Embedding in agarose
Tissue section (vibratome)
Heme removal (48 hours)
Immunostaining (5 to 10 days)
Clearing (24 hours)
Imaging
18 to 25 days
B.
Clearing
-
+
Calvaria
Femur
C.
IVM
Cleared
vWF - Dextran
vWF - Endmcn
D.
p=0.83
MGK diameter (μm)
IVM
Cleared Section
E.
30 min.
1 hour
2 hours
4 hours
6 hours
CD3
F.
Ratio of cell-to-background pixel intensity
Fixation time (h)
G.
i.
ii.
Pan-Laminin
H.
Pan-Laminin
i.
ii.

Figure S1

Figure S1: Bone marrow thick section preparation, related to Figure 1.
(A) Bone preparation workflow. Following harvest, the bones are cleaned, fixed for 2 hours and decalcified for 10-15 days. The bones are then embedded in 4% agarose and sectioned on a vibratome at a thickness of 250 µm. After heme removal (48h), sections are incubated with specific antibodies and cleared before imaging. **(B)** Comparison of the calvarium (left panels) and femur section (right panels) before and after clearing. **(C)** Effect of clearing protocol on bone marrow integrity. The same calvarium is imaged in vivo by intravital microscopy before undergoing the staining and clearing protocol and is re-imaged afterward. The left part of the image shows the IVM acquisition while the right side of the image display the post clearing acquisition. Blood vessels (green) are identified by FITC dextran intravenous injection in the IVM image and endomucin (Endmcn) immunostaining on the processed bone. MKs are identified thanks to vWF reporter transgene (red). **(D)** Quantification of MK size before (IVM) and after clearing protocol. **(E-F)** Effect of fixation time on immunostaining efficiency and background. **(E)** Representative images of each time point using the CD3 antibody (magenta). **(F)** The ratio cell to background is plotted. Scale bars = 50µm. **(G-H).** Blood vessels morphology after decalcification: lightsheet imaging of blood vessels identified via pan-Laminin immunostaining on a 250µm thick section prepared as described in (A). **(G)** Full femur image (scale bar = 1mm). Magnifications of bone lining marrow (i) and trabecular region (ii) are shown in **(H)** (scale bars = 500µm). The dashed lines indicate the bone determined by autofluorescence.

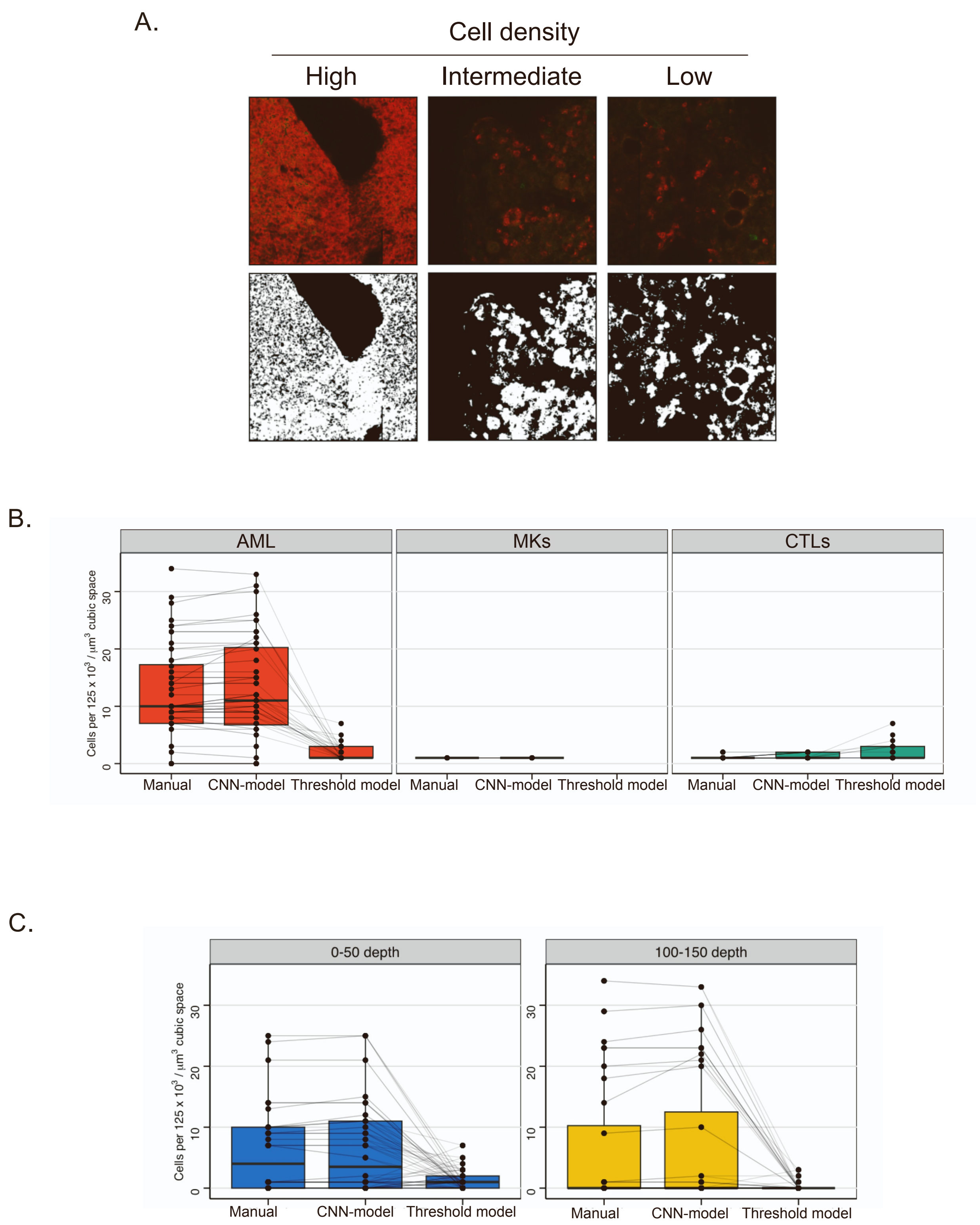

Figure S2: Comparison with threshold model of image analysis, related to Figure 1.
(A) Representative images showing the raw data (top) and the output (bottom) of the threshold model for AML cells. **(B)** Quantification of the number of cells identified using manual annotation, the neural network model and threshold model. **(C)** Comparison of each annotation model efficiency across different z-depths of the image.

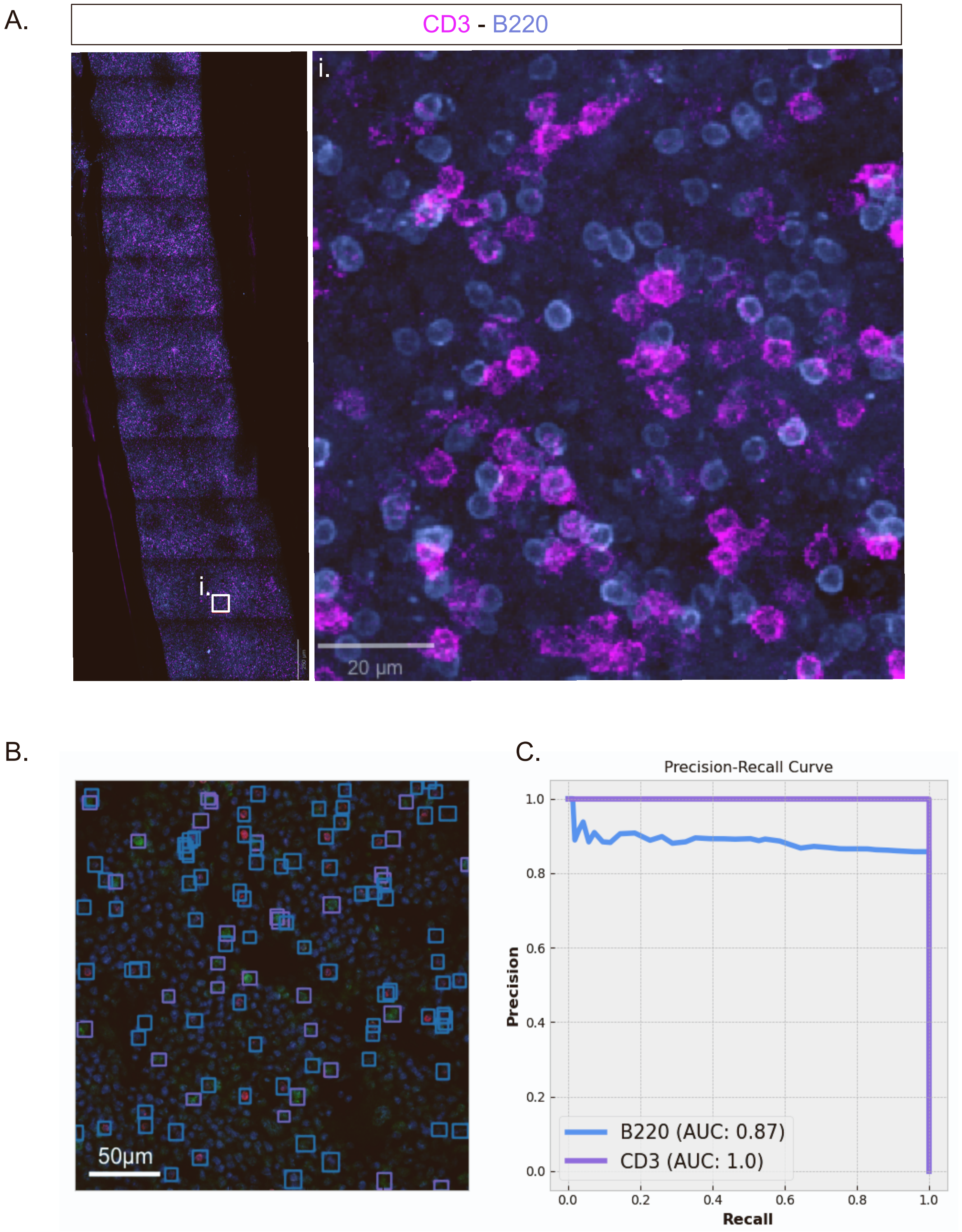

Figure S3: Neural network performance detecting bone marrow B and T cells, related to Figure 1. **(A)** Maximum intensity projection of five z-positions (z = 25 to 30 out of 39 total) of a 250 µm clarified mouse femur section. B ant T cells cell membranes are labelled by B220 and CD3 immunostaining respectively. Inset (i) shows a high-magnification image. **(B)** Representative example of B cell (blue boxes) and T cels (purple boxes) predictions generated by the 2D object detection. The model was applied without retraining from the version used in the main figures to detect MGKs, CTLs and AML cells **(C)** Precision-recall curve illustrating the performance of the 2D object detection model for each cell type.

A.

Moran's I

Cube size	AML cells	CTLs	MKs
45µm³	0.81	0.34	0.05
55µm³	0.82	0.45	0.09

B.

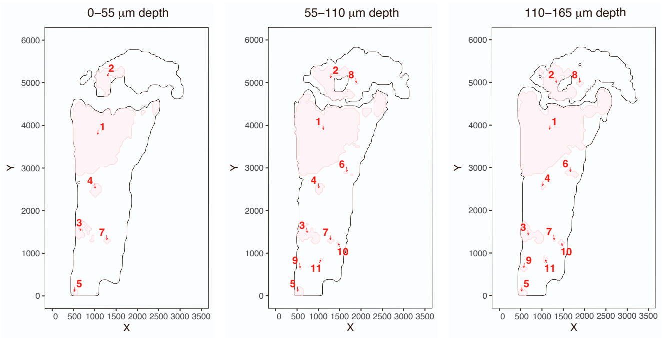

C.

$45µm^3$ cubes	Coefficient	Exp(coef)	p-value
Only CTLs	-1.58	0.2051	< $2x10^{16}$
CTLs and MKs	-1.57	0.2085	< $2x10^{16}$
Only MKs	-0.06	0.939	0.34

$55µm^3$ cubes	Coefficient	Exp(coef)	p-value
Only CTLs	-1.8	0.16	< $2x10^{16}$
CTLs and MKs	-1.8	0.16	< $2x10^{16}$
Only MKs	0.14	1.15	0.09

D.

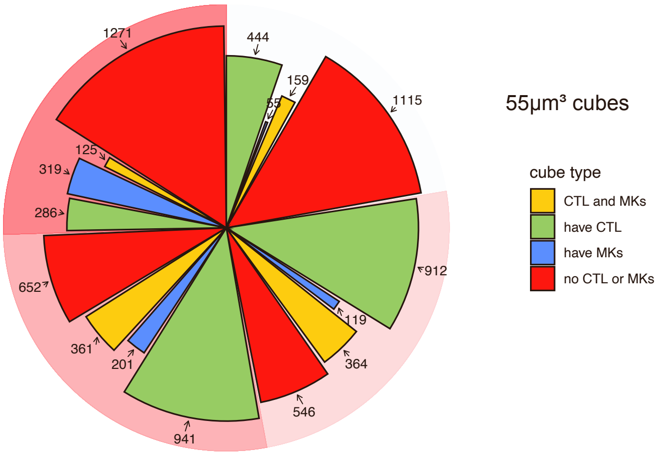

Figure S4: Effect of cube sizes on the spatial analysis, related to Figure 3.
(A) Table displaying the Moran's I values for various cube sizes. **(B)** 2D visualization of AML clusters identified by DBSCAN at a cube size set at 55µm3. The clusters are numbered in descending order based on the total AML cell count they contained. **(C)** Logistic regression results with the cube size of 45 µm3 (left) or 55 µm3 (right). **(D)** Pie chart showing the classification of 55µm3 cubes within each AML density quartile based on the presence of CTLs, MKs, or both.
