# Supplementary material for: Practical AI-based cell extraction and spatial statistics for large 3D bone marrow tissue images
Source: Cell Rep Methods. 2026 Mar 13;6(3):101334. doi: 10.1016/j.crmeth.2026.101334 (PMC13030987; doi:10.1016/j.crmeth.2026.101334)
Supplement: Document S2. Article plus supplemental information [file mmc2.pdf]

Cell Reports
Methods

Article

Practical AI-based cell extraction and spatial statistics for large 3D bone marrow tissue images

Graphical abstract

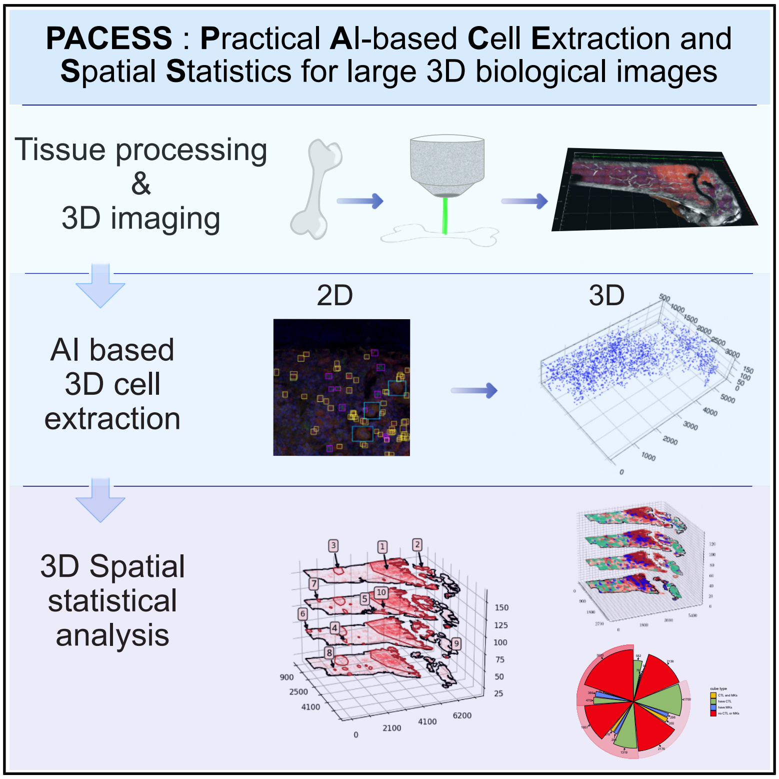

Highlights

- We develop PACESS, a method for generating and analyzing large 3D bone marrow samples
- PACESS provides the location of cells of interest and classifies cell types
- PACESS contains a statistical framework for identifying cell clusters
- PACESS identifies local heterogeneities within acute myeloid leukemia cell clusters

Authors

George Adams, Floriane S. Tissot, Chang Liu, Cera Mai, Chris Brunsdon, Ken R. Duffy, Cristina Lo Celso

Correspondence

k.duffy@northeastern.edu (K.R.D.),
c.lo-celso@imperial.ac.uk (C.L.C.)

In brief

Adams et al. develop PACESS, a method for generating and analyzing large 3D bone marrow sections. PACESS uses neural networks and spatial statistical approaches to classify hundreds of thousands of cells and reveal cellular relationships in bone marrow.

Adams et al., 2026, Cell Reports Methods *6*, 101334
March 23, 2026 © 2026 The Authors. Published by Elsevier Inc.
https://doi.org/10.1016/j.crmeth.2026.101334

CellPress

Cell Reports Methods

CellPress
OPEN ACCESS

Article

Practical AI-based cell extraction and spatial statistics for large 3D bone marrow tissue images

George Adams,[1,2,8] Floriane S. Tissot,[1,2,3,8] Chang Liu,[4,8] Cera Mai,[1,2,3] Chris Brunsdon,[5] Ken R. Duffy,[6,7,9,*] and Cristina Lo Celso[1,2,3,9,10,*]

[1]Department of Life Sciences, Sir Alexander Fleming Building, Imperial College London, London SW7 2AZ, UK
[2]Centre for Haematology, Department of Immunology and Inflammation, Imperial College London, London W12 0NN, UK
[3]The Francis Crick Institute, London WC2A 3LY, UK
[4]Hamilton Institute, Maynooth University, Maynooth, Co. Kildare, Ireland
[5]National Centre for Geocomputation, Maynooth University, Maynooth, Co. Kildare, Ireland
[6]Department of Electrical and Computer Engineering, Northeastern University, Boston, MA 02115, USA
[7]Department of Mathematics, Northeastern University, Boston, MA 02115, USA
[8]These authors contributed equally
[9]These authors contributed equally
[10]Lead contact
*Correspondence: k.duffy@northeastern.edu (K.R.D.), c.lo-celso@imperial.ac.uk (C.L.C.)
https://doi.org/10.1016/j.crmeth.2026.101334

MOTIVATION The high cellular density of bone marrow (BM) complicates image segmentation, and current spatial analyses are often restricted to pairwise comparisons, unsuitable for investigating interactions between more than two cell types simultaneously. To overcome these limitations, we developed PACESS, a readily applicable neural network-based framework that classifies hundreds of thousands of cells in 3D BM samples and applies spatial statistical methods to evaluate multicellular interactions.

SUMMARY

Although the molecular regulation of hematopoiesis is well characterized, the spatial organization of hematopoietic cells within bone marrow (BM) remains unclear. Advances in microscopy have produced increasingly detailed images of murine BM, yet accurate and scalable methods to extract and analyze these complex datasets are limited. We present PACESS, a computational workflow for BM analysis that combines convolutional neural networks for 2D cell detection and classification with an automated method to extrapolate into 3D, spatial statistical analyses to define tissue regions based on local cell-type densities, and logistic regression to assess whether the relative abundances of cell types reflect reciprocal dependencies. Using PACESS, we investigate the spatial organization of T cells, megakaryocytes, and leukemic cells, revealing that distinct leukemic clusters generate diverse, previously unrecognized neighborhoods within the same BM cavity. PACESS, thus, provides a powerful tool to dissect BM architecture.

INTRODUCTION

The spatial architecture of tissues is a key determinant of organ function, and disruptions in cellular organization are often linked to diseases. This has been extensively demonstrated for structurally defined tissues such as the skin or the brain.[1,2] Bone marrow (BM) is the primary site of hematopoiesis, where billions (in mice) to trillions (in humans) of diverse blood cells are generated daily. The combination of flow cytometry, single cell transcriptomics, and functional assays has provided a wealth of information on the different hematopoietic cell populations co-existing in the BM.[3–5] However, still very little is understood about the spatial organization of this tissue. BM is the softest tissue in the body and has long been considered as an amorphous, even liquid, structure with numerous cell types tightly packed within a confined cavity. These features make the BM uniquely challenging for histological imaging and quantification. To overcome this, recent efforts have employed increasingly sophisticated BM multidimensional imaging techniques (3D or greater), focusing on visualizing hematopoietic stem and progenitor cell (HSPC) populations in large tissue volumes.[6–8] While those methods led to the emerging concept that the BM is spatially and regionally organized to differentially support distinct HSPC populations,[6,9] the reciprocal spatial relationships between multiple hematopoietic cell types remain unclear. Better understanding would hold clues for more effective harnessing of HSPC function, development of improved therapies for hematological disorders, and overall healthier aging.

© 2026 The Authors. Published by Elsevier Inc.
This is an open access article under the CC BY license (http://creativecommons.org/licenses/by/4.0/).

Quantification of multicellular interactions within large 3D BM image datasets has not yet been achieved due to two main technical limitations. First, image segmentation by intensity thresholding, which is historically the most widely used method for identifying cells of interest,[9–12] underperforms in tissues such as the BM where cells are tightly packed together and boundaries between cells become difficult to distinguish. Hence, a large number of cells are not identified and are lost. Additionally, segmentation-based approaches can be computationally demanding[9] and tend to be impractical when dealing with very large images such as the 3D preparations needed to capture the overall organization of cell types across BM tissue.[13] Some deep learning-based methods have been developed but utilized primarily for the identification of BM vasculature and single cell types with highly consistent morphology.[14,15] The second challenge relates to the statistical methodologies available to assess multicellular interactions. Existing statistical approaches are typically restricted to pairwise comparisons, limiting the ability to assess higher-order interactions among multiple cell types and to draw comprehensive conclusions from complex datasets.[6,10–16]

To address these challenges, we present PACESS (practical AI-based cell extraction and spatial statistics), a pipeline for extracting and analyzing hematopoietic cells from large, 3D BM images (graphical abstract). PACESS makes use of convolutional neural network (CNN)-based object detection to classify and identify the locations of cells in 2D, followed by an automated method that extrapolates cellular localization into 3D. We employ an augmented object detection deep neural network trained solely on 2D annotations, enabling rapid labeling and, therefore, scalable deployment. Annotations are created for each image layer in the 3D data, and the outputs from multiple layers are automatically combined to determine each cell's identity and 3D spatial coordinates. Once the spatial data are extracted, we apply a set of 3D spatial statistical analyses including: (1) exploratory statistics to assess spatial heterogeneity; (2) identification of regions of high cellular density of specific cell types; (3) visualization of local cellular densities; and (4) inference of location-dependent relative abundances among cell types. These steps culminate in a comprehensive statistical map of the BM microenvironment.

We applied PACESS to study the spatial interactions of megakaryocytes (MKs) and T cells in the context of an acute myeloid leukemia (AML) *in vivo* model. AML is known to disrupt normal functioning and organization of the BM, including the dislodgment of healthy hematopoietic cells,[17,18] and, therefore, represents a good model for studying changes in multiple cell populations' spatial interactions. First, PACESS was able to efficiently extract the cellular information for the three cell populations, even in areas of high AML density. Next, the statistical analysis revealed that the local AML burden at specific BM sites significantly affected T cells' and MKs' densities, albeit with distinct patterns. This led to the identification of discrete microenvironments, each characterized by unique local compositions of these three cell types. Together, these findings demonstrate that PACESS enables the systematic mapping of cellular organization in complex tissues and offers a scalable approach to dissecting multicellular interactions in homeostasis and disease.

RESULTS

Large, 3D image datasets of murine BM represent a computational challenge for analysis

The BM is an organ where cells are highly packed together, and this is exacerbated during AML, when malignant cells expand and outcompete healthy hematopoietic cells through mechanisms that remain incompletely understood. Investigating the spatial relationships among BM cell types requires imaging large, intact 3D tissue volumes. We generated these using agarose embedding and a vibratome to cut 250-μm thick sections of various bones. We established a robust and reproducible BM-clearing protocol by integrating and systematically optimizing elements from previously published methods for BM and other tissues. Through iterative refinement and careful monitoring of clearing endpoints—including extensive troubleshooting of fixation parameters—we fine-tuned the workflow to ensure consistent tissue transparency and structural preservation (Figure S1).

Heme is highly abundant in the BM and is known to interfere with light penetration, generating background noise and thus reducing image quality. We first used the Reagent 1 (or ScaleCUBIC1) solution from the CUBIC method to decolorize heme efficiently.[19] To quickly clarify BM samples while maintaining their integrity over time, we selected the Ce3D tissue clearing reagent[20] and kept the samples in it for imaging and storing (Figures S1A and S1B). To verify that our clearing protocol preserved native BM architecture, we first performed intravital two-photon microscopy (IVM) of calvarial BM of a vWF:tomato MK transgenic reporter mouse without any clearing, and subsequently, after tissue harvesting and clearing, we imaged the same bone *ex vivo*. The mouse was injected with FITC-conjugated dextran to identify vasculature during IVM, and the calvarium sample was stained with anti-endomucin antibody to achieve the same result *ex vivo*. Juxtaposition of the tilescans obtained via IVM and following clearing (Figure S1C) revealed no changes in the overall appearance of the tissue. Moreover, MK diameters were unchanged between the pre- and post-clearing images (Figure S1D). In a separate test, we confirmed that vascular morphology remained intact across the entire tissue (Figure S1G), including in bone-lining and trabecular regions (Figure S1Hi and S1Hii, respectively).

Finally, to maximize the signal-to-noise ratio in thick BM sections, we systematically evaluated the effects of fixation on antibody staining quality. Fixation duration had antibody-specific effects, with more antibodies resulting in a poor signal-to-noise ratio the longer fixation would last. Overall, 2-h fixation emerged as the optimal condition, striking a balance between preserving tissue for efficient and reliable vibratome sectioning and maintaining strong, interpretable immunostaining (Figures S1E and S1F).

To generate an exemplar image, we injected a wild-type C57Bl/6 mouse with tomato^{+} AML cells generated through retroviral transduction of the oncogene MLL-AF9, a well-established and widely used murine model of AML.[21,22] We harvested bones at an intermediate disease stage, at which point flow cytometry analysis of one femur confirmed an overall BM infiltration of 15%. A 250-μm thick section from the contralateral femur was stained

CellPress
OPEN ACCESS

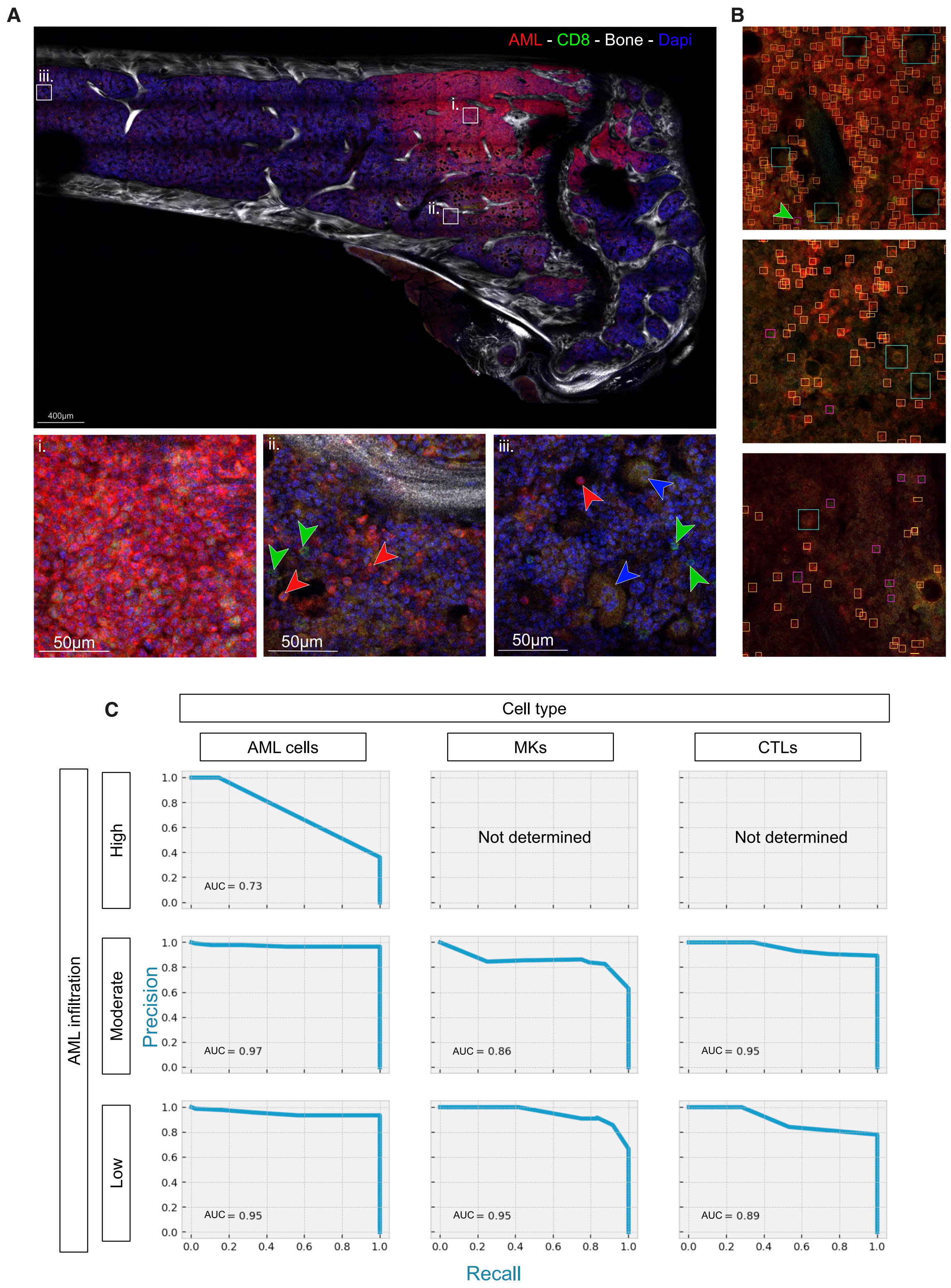

Figure 1. 2D data extraction using the deep neural network

(A) Tile scan of a single z-position (z = 27 out of 32) from a clarified 250-µm thick tibia section of an AML-infiltrated mouse. CTLs' cell membranes are labeled by CD8 immunostaining (green), AML cells express membrane-targeted tdTomato (red) and low level of intracellular GFP (green), nuclei are counterstained using DAPI (blue), and bone is visualized using SHG (gray). MKs are identified based on their distinct morphology, characterized by their large size, multilobate nuclei,

(legend continued on next page)

with an anti-CD8 antibody, followed by species-specific fluorophore-conjugated secondary antibody and DAPI nuclear counterstain to highlight cytotoxic T lymphocytes (CTLs), cleared and imaged using a combination of confocal and two-photon microscopy. In the resulting 3D tilescan image, bone was visualized through second harmonic generation (SHG) signal of collagen (represented in grayscale); AML cells were identified by membrane-localized tomato fluorescence (red), CTLs by CD8 immunostaining (green), and MKs by their large size, multilobated nuclei, and characteristic autofluorescence (pale green/red). Nuclei of all cells were labeled with DAPI (blue) (Figure 1A).

An initial visual inspection of the image confirmed an uneven distribution of AML cells, whereby the BM region proximal to the growth plate exhibited dense leukemic infiltration, with tomato$^+$ cells tightly packed together (Figure 1Ai), whereas more distal regions showed variable levels of local AML infiltration (Figure 1Aii and 1Aiii). However, visual analysis alone was insufficient to reliably quantify local variations in CTL and MK density or to determine whether the abundance of one cell type was dependent on the density of another. Addressing these questions is critical to understanding how AML cells reshape the BM microenvironment during infiltration and requires accurate, high-resolution identification and annotation of all relevant cell types.

Data extraction using deep CNN

The raw image (as seen in Figure 1) was 15 GB in size and was the result of juxtaposition of 33,775 partially overlapping 2D images acquired as adjacent z stacks. In total, the image was made of 32 layers of 493 tiles sized 512 $\times$ 512 pixels, with separation distance between the layers (z-step) being 5 μm. Conventional image analysis software is typically unable to open or process entire datasets of this scale and lacks the capacity to efficiently handle large volumetric images. To address this, we implemented a YOLO (You Only Look Once) CNN model that could be trained on small, 2D image patches randomly sampled from the full tilescan, operate on image subsets, and output lightweight prediction files that are computationally tractable.[23,24] In the image presented in Figure 1A, we manually annotated a total of 18,240 cell examples, of which 14,655 were AML cells, 3,056 were CTLs, and 529 MKs. Annotations were divided into training (70%), validation (20%), and testing (10%) datasets. The model was trained for 200 epochs, and the optimal performance was achieved at epoch 60. The final trained network was used to identify 506,611 cells across all 2D slices using bounding boxes, which remained visually interpretable (Figure 1B).

To evaluate model performance, we manually classified all test images according to whether they represented areas of high, moderate, or low AML infiltration (Figure 1Ai, 1Aii, and 1Aiii, respectively) and compared our manual annotation with the results of the model to generate precision-recall curves (Figure 1C). The model performed least accurately in regions of high leukemic infiltration, where densely packed AML cells were somewhat under-identified (Figure 1C, top left), although the total number of AML cells returned remained high (e.g., Figure 1B, top). In contrast, CTLs and MKs were reliably identified, even in heavily infiltrated areas (Figure 1B), but their low abundance limited the statistical power for a precision-recall analysis (Figure 1C, top center and top right). In regions with intermediate or low leukemic burden, the model performed consistently across all three cell types, achieving a mean AUC of 0.8942 (Figure 1C). Individual AUCs for AML cells, CTLs, and MKs are shown in the corresponding panels. In addition to benchmarking against manual annotation, we compared our CNN to a conventional, well-established multi-level Otsu thresholding model[25,26] on 3D samples selected to include densely, intermediate, and sparsely infiltrated areas. The network model achieved results that most closely matched manual annotation (Figures S2A and S2B), with equivalent accuracy across the depth of the image (Figure S2C), while thresholding clearly underperformed in comparison to CNN, and manual annotation and was not applicable to MK annotation (Figures S2B and S2C).

An advantage of CNN models is that they can be easily adapted for other images. To test whether our CNN model could identify other cell types similar to CTLs and to evaluate how much additional training would be required, we applied it to the image of an independent sample immunostained with anti-B220 and anti-CD3 antibodies, highlighting B cells and all T cells (Figures S3A and S3B). Manual benchmarking indicated that, without any additional training, the CNN achieved an optimal precision-recall curve for CD3 T cells, a heterogeneous population that includes CTLs, and an excellent curve for B cells (Figure S3C). In conclusion, the model was able to identify cells in 2D images based on a combination of fluorescent signals and morphological features and could easily be implemented for the analysis of additional cell types.

To generate maps of the 3D locations of cells across the entire dataset, we developed a custom 3D aggregation algorithm that linked adjacent bounding boxes across image layers. This was an important simplification of the workflow because these 3D maps could be generated without the need for 3D labeled training data. The resulting 3D boxes could then be overlaid onto sub-volumes of the original dataset for visual inspection and validation (Figures 2A–2C). To assess the accuracy of the 3D reconstruction step, we randomly selected 3 separate 210 $\times$ 210 $\times$ 50 μm^3 3D regions with high, medium, and low AML infiltration within the overall image, manually annotated AML cells, CTLs, and MKs in 3D, and compared the manual and predicted annotations (Figure 2D). The predicted cell counts

and autofluorescence. (i–iii) High magnification images of specific regions with (i) high, (ii) intermediate, and (iii) low AML infiltration. Arrows indicate representative AML cells (red), CTLs (green), and MKs (blue).

(B) Example of 2D object detection predictions for AML cells (yellow boxes), CTLs (purple boxes), and MKs (blue boxes). Shown are representative images from areas with high (top), intermediate (middle), and low (bottom) AML infiltration. Green arrow highlights a CTL identified in an area of dense AML.

(C) Precision-recall curves of the 2D object detection model for each cell type, stratified by regions of high (top), intermediate (middle), or low (bottom) AML infiltration.

AML, acute myeloid leukemia; CTLs, cytotoxic T lymphocytes; MKs, megakaryocytes; SHG, second harmonic generation.

See also Figures S1, S2, and S3.

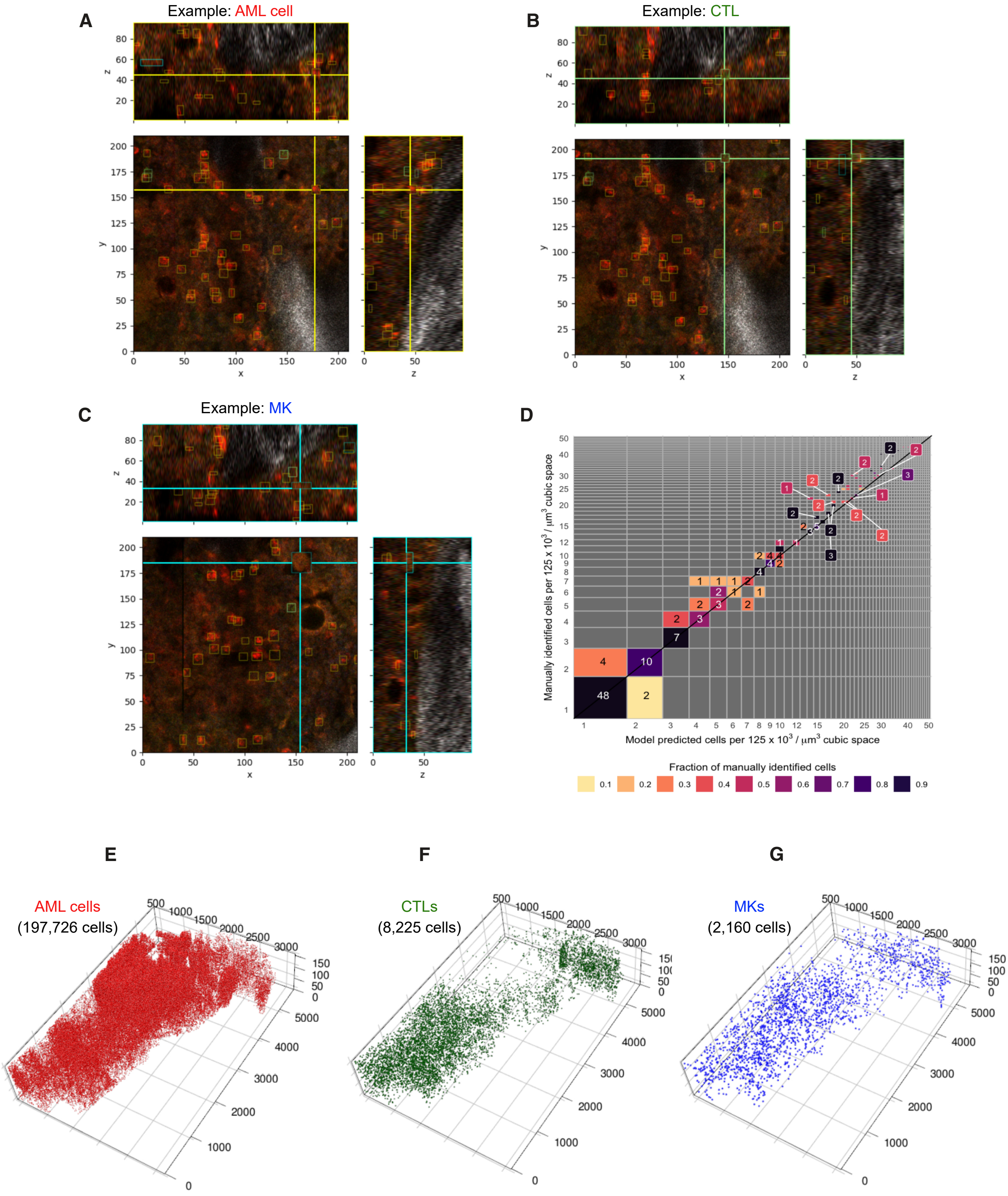

Figure 2. 3D reconstruction of the bone marrow cellular organization

(A–C) Representative images showing 3D bounding boxes for (A) AML cells (yellow boxes), (B) CTLs (green boxes), and (C) MKs (blue boxes). AML cells express membrane-targeted tdTomato (red) and low level of intracellular GFP (green), CTL membranes are labeled by CD8 immunostaining (green), and MKs are identified based on their specific cellular morphology (large size, multilobate nuclei, and autofluorescence). Scales are in μm.

(legend continued on next page)

closely matched manual annotations, with only a slight underestimation at higher cell densities. Using the automated approach, we identified 197,726 AML cells, 8,225 CTLs, and 2,160 MKs, numbers that would be impractical to obtain manually. In addition to cell counts, the algorithm preserved precise spatial localization for each cell (Figures 2E–2G).

Visualization of the full 3D reconstruction revealed that relying on 2D sections alone underestimates the AML burden, even in regions that appear minimally infiltrated (Figure 2E). Consistent with previous reports of healthy hematopoietic cells being excluded from highly infiltrated areas of BM tissue,[17,22] the area near the growth plate appeared devoid of CTLs (Figure 2F). In contrast, MKs displayed a distinct distribution pattern from CTLs (Figure 2G), underscoring the need for a dedicated spatial analysis pipeline capable of resolving cell type-specific patterns and their relationships within complex tissue environments.

Spatial heterogeneity and automated identification of areas of high cellular density

To visualize and quantify the spatial distribution and interrelationships between AML cells, CTLs, and MKs, we discretized the 3D imaging volume into adjacent, non-overlapping cubes. These cubes served as spatial units for downstream statistical analysis and were used first to quantify heterogeneities in the distribution of cell types, and next to explore the cells' interdependencies. To assess spatial heterogeneity, we used two steps: first, we used Moran's I (also known as spatial autocorrelation function)[27] to assess the similarity between neighboring cubes; next, we linked similar cubes to outline areas of high cellular density, using the density-based spatial clustering of applications with noise (DBSCAN) algorithm[28] (Figure 3A).

We first determined an ideal cube size to balance spatial resolution with computational tractability. For meaningful visualization, the discretization needs to be such that some aggregation of cell counts occurs, but a few cells of the same type remain in each cube. A cube size of 45 μm^3 was selected to be sufficiently fine that spatial resolution could be retained, but sufficiently coarse that the resulting data could still be computationally assessed without undue burden. Using this discretization, we recorded the number of AML cells, CTLs, and MKs in each cube (Figure 3A, left), enabling spatial visualization of their densities across the tissue volume (Figure 3B). This information was the basis to quantify spatial heterogeneity both within and between cell populations.

To quantitatively assess the homogeneity of each cell type's spatial distribution, we calculated their Moran's I. For that, one must consider the similarity between each spatial unit (one of the cubes) and its neighbors. Neighbors can be defined in several ways. In our framework, two cubes were considered neighbors if they shared a face, resulting in a 7-cube neighborhood per central cube (Figure 3A, center) with smaller neighborhoods for cubes adjacent to a boundary. Moran's I is a measure of the overall homogeneity of cell numbers in cubes across the tissue with reference to their neighbors. If Moran's I is positive, cells tend to be aggregated in common areas. If Moran's I is close to zero, cells are distributed randomly in space. When Moran's I value is less than zero, cells are more homogeneously dispersed than one would expect from a random process. Moran's I for the AML cells was 0.81, quantitatively substantiating the observation that AML cells were concentrated in relatively few areas. Moran's I for CTLs was 0.34, suggesting some positive spatial clustering but less than that found for AML cells. Moran's I of 0.05 for MKs indicated these cells had a weak spatial dependency, resembling that of random locations (Figure 3B).

While Moran's I can indicate whether cells of a given type are overall co-located or scattered throughout the tissue, it does not identify tissue regions where those cells are at high density. We, therefore, used the DBSCAN algorithm[28] to identify spatial clusters. A neighborhood of cubes was defined as dense if the average number of cells in its cubes was greater than the third quartile value for that population in all cubes across the sample, whereby only a quarter of cubes have more cells than this value. This calculation would be repeated by shifting the neighborhood center from one cube to the adjacent one, until all possible neighborhoods would be considered. As a result, dense neighborhoods were agglomerated to highlight contiguous areas or clusters (Figure 3A, right).

DBSCAN identified 22 distinct AML high-density areas. The largest 10 are marked in decreasing order, from the largest to the smallest (Figure 3C). The largest cluster (cluster 1) accounted for 61.8% of all AML cells in the image, and clusters 2 and 3 for 5.9% and 2.0% of all AML cells, respectively. The two largest clusters of AML cells, 1 and 2, were proximal to the growth plate, on either of its two sides. Cluster 3 was more distal, and overall, the distal region of the BM contained much smaller clusters. Interestingly, larger clusters were located adjacent to the endosteum, whereas centrally located clusters were generally smaller. For CTLs and MKs, approximately 70% and 90% of cubes, respectively, recorded a cell count of zero (Figure 3A, left), and, consequently, DBSCAN returned no clustered areas for these populations. This was consistent with CTLs and MKs being less abundant than AML cells, generally not clustered, and returning Moran's I values that were positive but close to zero.

Spatial dependencies between cell types

Understanding the principles that govern multicellular tissue organization requires insight into how diverse cell types interact with each other to enable tissue function. A key question is whether local heterogeneities in the distribution of a given cell type are influenced by the presence or abundance of others, so that the resulting different cellular neighborhoods can be identified and mapped. To address this, we first assessed whether, besides low Moran's I and lack of clustering, CTLs and MKs localization could be described as a function of AML cell presence and/or density. To investigate this, we used a

(D) Comparison of manually identified versus model-predicted cell identification per 125 × $10^3/\mu m^3$ cubic space.
(E–G) 3D reconstruction of AML cells (E), CTLs (F), and MKs (G) within the bone marrow. The total number of identified cells for each population is indicated in black.
AML, acute myeloid leukemia; CTLs, cytotoxic T lymphocytes; MKs, megakaryocytes.

CellPress
OPEN ACCESS

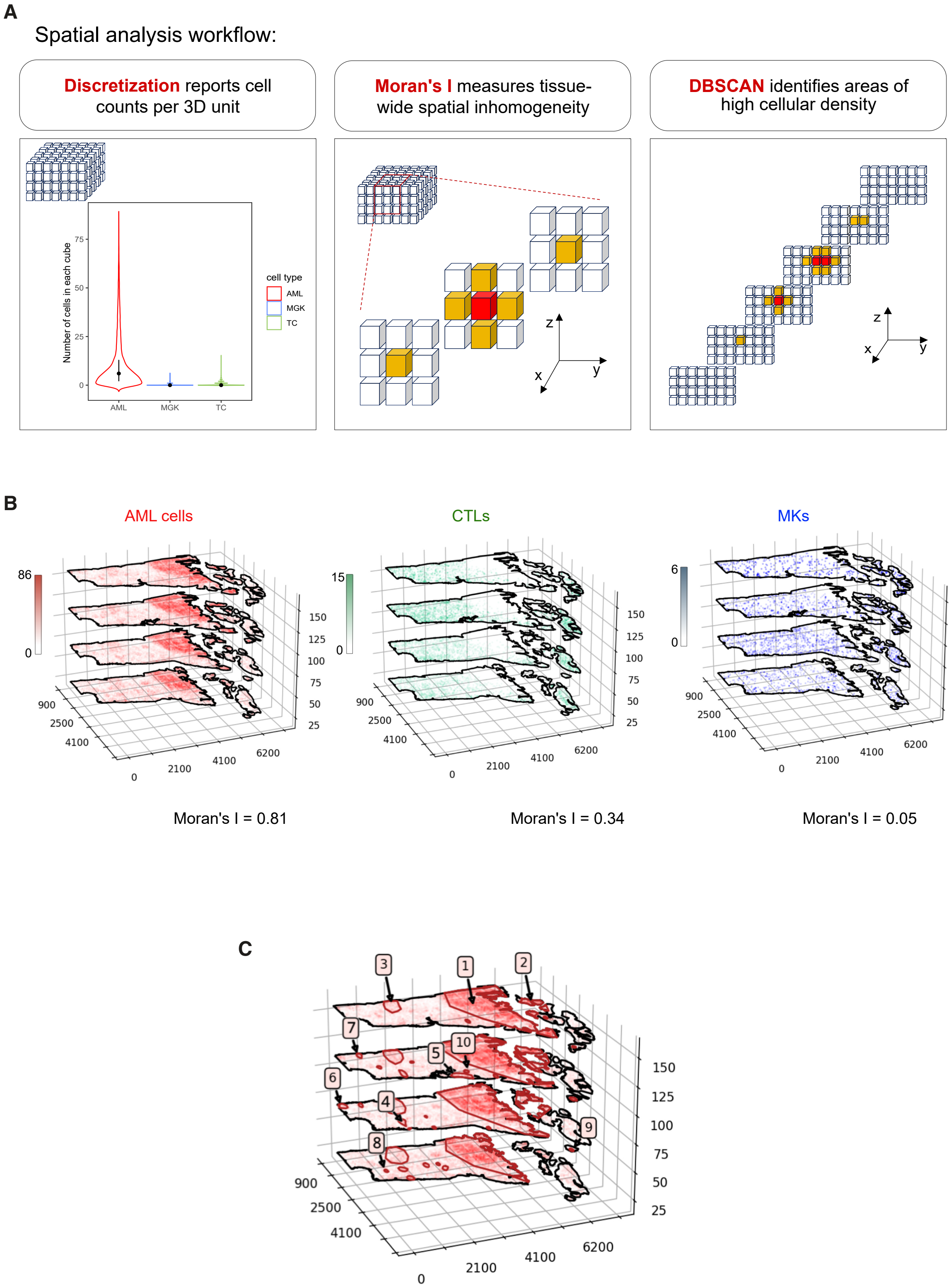

Figure 3. Spatial analysis framework for quantifying cell distribution and clustering

(A) Overview of the spatial analysis framework. Space is discretized into cubic units, allowing the measurement of local cell densities. Moran's I measures the spatial autocorrelation of each cell type by assessing whether similar cell densities are clustered together or randomly distributed. In the dataset, each cube (red)

(legend continued on next page)

combination of permutation tests and logistic regression (Figure 4A).[29]

For CTLs, a visual inspection of the image indicated that their density was uneven throughout the tissue, with marked exclusion of CTLs from the area corresponding to AML cluster 1, (Figures 2E, 2F, 3B, and 3C). To statistically assess if the distribution of CTL counts was influenced by any areas of high AML density, we used permutation tests to challenge the null hypothesis that the mean number of CTLs within each cube is independent of whether the cube is within an AML cluster or adjacent to it (Figure 4A, left panel). The null hypothesis that CTL density is independent of AML cluster proximity was rejected only for AML cluster 1 (Figure 4B). The remaining 21 smaller AML clusters all failed to reject the null hypothesis. This analysis indicated that only the largest AML cluster substantially impacted CTL spatial distribution by influencing the mean number of CTLs per space unit. In other words, CTLs were generally missing where the largest AML cluster was located, but their density was not affected in the smaller areas with dense AML cells.

We next applied the same spatial permutation strategy to assess MK distribution. The results showed no evidence that MKs had a different distribution within versus around any AML dense area (Figure 4C). However, a visual inspection of the image highlighted heterogeneity with the AML cluster 1 region, which could be further split into two sub-regions that either lacked or retained MKs. This suggested the presence of more nuanced spatial dependencies that warranted additional analysis.

To further examine the likelihood of CTLs and MKs being present alone or in combination in AML-infiltrated areas, we used logistic regression. This statistical model reports on the likelihood of an event to occur or not given a certain binary condition, in our case the presence of cubes containing CTLs, MKs, or both inside vs. outside dense AML areas (Figure 4A, right panel). The probability of finding cubes with CTLs or both CTLs and MKs inside dense AML areas was 80% lower than that of finding such types of cubes outside of dense AML areas (both p values <2e-16). The likelihood of finding cubes with MKs but not T cells inside dense AML areas was lower, but not significantly, than that outside of them (Figure 4D). For MKs, this latter result was driven by heterogeneity within AML high-density area 1, where one subregion was entirely depleted of MKs, while an adjacent subregion contained MKs at higher density than the rest of the tissue (Figure 4D, right panel, black arrow vs. blue arrow).

To examine whether AML density alone could predict MK presence, we developed a visualization tool in which all cubes were binned into quartiles based on AML cell density (Figure 4E). Each cube was further classified according to the presence or absence of CTLs and/or MKs, yielding 16 possible combinations of the neighborhood composition. Although all 16 cell type combinations were present, some were more abundant than others, with areas containing both CTLs and MKs being the rarest, independent of the AML density quartile (Figure 4F). Spatial visualization of the neighborhoods highlighted tissue architecture heterogeneities. Outside of dense AML area 1, various types of neighborhoods could be found adjacent to each other, without a specific pattern (Figure 4G). The dense AML cluster 1 was split into two regions based on MK presence. The sub-region closest to the growth plate was generally devoid of MKs, and the MK-rich sub-region was more distal, but the two sub-regions were not simply juxtaposed because MK-containing areas were most often surrounded by MK-depleted areas, forming discontinuous pockets of MK-rich tissue (Figure 4H). Both inside and outside AML cluster 1, the MK-rich and CTL-rich areas were irregularly shaped, with more jagged contours than AML dense areas, indicating that while regional variations in the tissue composition could be identified, these regions had irregular and intertwined shapes.

Considerations on PACESS pipeline implementation

To assess whether the results of spatial analyses would be strongly influenced by the cube size used, we re-analyzed the image using larger cubes sized 55 μm^3, instead of 45 μm^3. The results obtained were universally consistent, with AML cells continuing to return the highest Moran's I and MKs the lowest (Figure S4A). DBSCAN identified 11 AML high-density clusters that overlapped with the 11 largest ones found with the 45 μm^3 cube size (Figure S4B), indicating that larger cube sizes would lead to the loss of the smallest clusters. Permutation tests and logistic regression test results remained essentially unchanged (Figure S4C), and so did the distribution of the neighborhood types (Figure S4D). Smaller cube sizes would lead to the resulting datasets increasing at the cubic scale, and therefore, rapidly becoming computationally more demanding. This analysis highlights the importance of choosing a cube size appropriate for the questions that a user would want to address while working with a computationally accessible dataset. As a result, rather than striving to identify an absolute quantification of any specific parameter, PACESS delivers a robust summary evaluation of the relative properties of multiple cell types co-existing within a tissue.

Finally, to test the robustness of our entire pipeline, we applied it to a second sample harvested from an AML-burdened mouse. Flow cytometry analysis of the hip bone indicated a slightly higher level of AML infiltration (48%, compared to 15% for the mouse used to generate the exemplar image shown in Figures 1, 2, 3, and 4). The sample and resulting images were

has adjacent neighbors (yellow), and Moran's I incorporates that local spatial information to calculate a global measure of spatial autocorrelation. DBSCAN is applied to identify high-density cellular regions. A neighborhood of cubes was defined as dense if the average number of cells in its cubes was greater than the third quartile value for that population in all cubes across the sample. Adjacent high-density regions are merged into larger contiguous clusters.

(B) Quantitative 3D visualization displays the spatial distribution of AML cells, CTLs, and MKs, with color scales indicating a cube's cell abundance. Moran's I values are reported for each cell type.

(C) 3D projection visualization shows AML clusters identified by DBSCAN. The clusters are numbered in decreasing order based on the total AML cell count they contained. The 10 first clusters are labeled on the image.

DBSCAN, density-based spatial clustering of applications with noise; AML, acute myeloid leukemia; CTLs, cytotoxic T lymphocytes; MKs, megakaryocytes. See also Figure S4.

CellPress
OPEN ACCESS

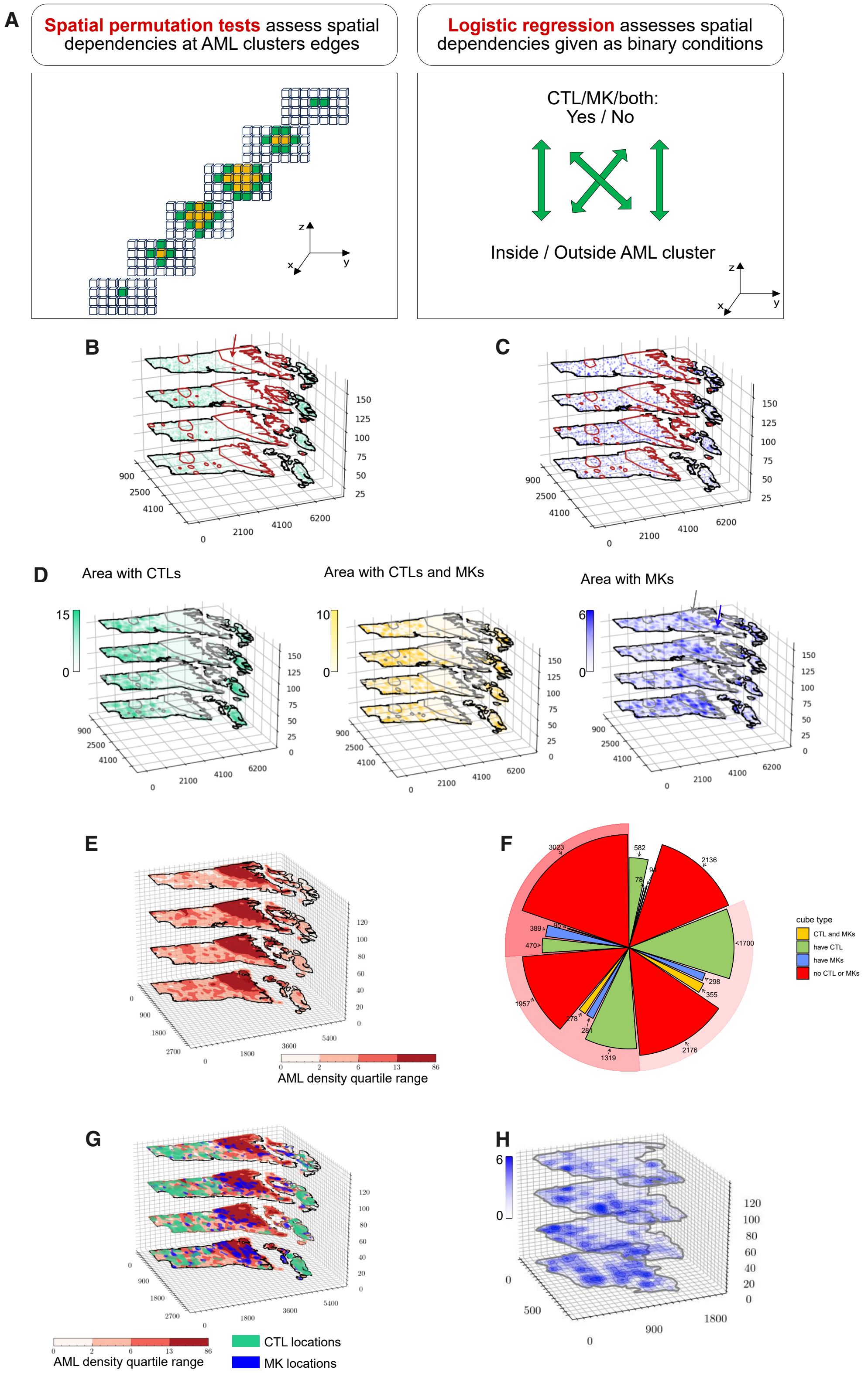

(legend on next page)

larger than the exemplar ones, spanning the entire tibia, and totaling 202,495,953 Kb for 12,236 tiles, covering 16.86 mm × 5.74 mm × 92 slices. The CNN identified 610,845 AML cells, 21,023 CTLs, and 4,420 MKs (Figures 5A and 5B). Spatial analyses yielded broadly equivalent results: AML cells were located in clusters, T cells were excluded from the densest AML clusters (Figures 5C and 5D), and MKs were increasingly enriched in the cubes containing more AML cells (Figure 5D). These results indicated that PACESS could highlight consistent patterns of local heterogeneities in BM samples partially infiltrated by AML cells.

These analyses, together with the ability of the CNN model to identify B cells and all T cells (Figure S3), demonstrate the reliability of the approach across different samples and experimental conditions.

In summary, the pipeline enables 3D spatial statistical analysis of large tissue datasets, including the quantification of clustering, identification of high-density regions, and evaluation of intercellular dependencies. Applying this framework, we found that only large AML-dense regions are significantly depleted of CTLs and that these regions can be further subtyped based on MK content. The irregular and intertwined boundaries between neighborhood types underscore the complexity of spatial tissue organization during leukemic infiltration.

DISCUSSION

In this study, we present PACESS, a novel, efficient, and scalable pipeline for extracting biological meaning and performing spatial inference on the distribution of cell types from large, 3D BM imaging datasets. Although such datasets are increasingly affordable to generate, they remain difficult to analyze due to their size and complexity. Quantitatively assessing the spatial relationships between distinct cell types remains a major challenge. However, this type of analysis holds substantial promise for elucidating the organizational principles regulating hematopoiesis, with important implications for therapeutic development and disease prevention in hematological disorders.

In terms of tissue preparation for image acquisition, we refined the optimal combination of tissue fixation, clearing, and mounting. The choice of clearing and mounting solutions was critical for maximizing tissue integrity over time. While previously described protocols resulted in tissue darkening and loss of fluorescent signals within a few days,[7,11,16] our optimized approach preserved signal quality and allowed reimaging of samples weeks apart. Fixation time also proved to be a key determinant of image quality. While various fixation times, from few hours to days, have been used across studies employing large BM specimens,[7,10,11] we found that a brief 2-h fixation step struck the optimal balance, enabling effective vibratome sectioning and achieving high signal-to-noise ratios. Compared to methods based on whole bone[30] or whole-mount[6,10,12] processing and on *in vivo* antibody injection-based immunolabelling,[30] PACESS enables independent immunolabelling of up to four different samples from a single long bone. Hence, multiple hypotheses on cell type interactions can be tested from a single bone, reducing the number of animals required for a study.

The images presented here are 3D composites of up to 15,776 2D image tiles, resulting in >4 × 10^9 pixels and up to 200 Gb large files that could not be opened using standard open access image analysis software such as ImageJ. Segmentation-based approaches have either been applied to images thinner than the one presented here[7,9,11] or focused on single cell types, often studied in relation to vasculature or bone signal,[9,15,30] and resulting spatial analyses have been used for testing binary hypotheses.[7,10–12] Our image analysis workflow builds on established techniques from machine learning and geospatial statistics and has several advantages over current approaches. From the perspective of data extraction, the workflow is reliable and scalable and, importantly, solves the challenge of efficiently labeling cell types in 3D by leveraging 2D annotation followed by 3D inference. The spatial statistics workflow allows identifying areas where any cell type of interest is denser than average and highlights tissue regions with unique cellular composition.

While AI-based extraction methods have been applied in other contexts, murine BM tissue provides distinct challenges because hematopoietic cell types are tightly packed together in an amorphous structure where no clear patterns have been identified. Deep learning approaches based on 3D training have been used to label, classify, and measure BM vasculature.[14,15] These models present challenges when dealing with variable cell types, which we resolved by utilizing 2D training.

Figure 4. Spatial relationships between cell types

(A) Left: for each cluster of cubes (yellow), the surrounding cubes (green) are identified. Spatial permutation tests are then conducted to test whether the mean density of a second cell type is statistically the same between the cluster and its surrounding region. Right: Logistic regression is performed to assess whether the presence or absence of other cell types is independent of dense AML areas.

(B) 3D projection visualization of hypothesis test results for CTLs. The null hypothesis is rejected for the largest AML cluster (number 1 indicated by the arrow) with Holm-Bonferroni corrected $p = 0.00007$.

(C) 3D projection visualization of hypothesis test results for MKs. Null hypothesis is not rejected, including in the largest AML cluster, with p values > 0.05 after Holm-Bonferroni correction.

(D) The results of logistic regression modeling, showing the likelihood of finding CTLs (left), CTLs and MKs (middle), and MKs (right) within dense AML regions. CTLs' p value is < 2e-16; CTLs' and MKs' p value is < 2e-16; MKs' p value is 0.464. Color shading indicates cell density. Arrows highlight MK-rich (blue arrow) or MK-devoid (gray arrow) areas within dense AML cluster 1.

(E) 3D projection visualization of cubes labeled based on quartile distribution of AML density.

(F) Pie chart showing the classification of cubes within each AML density quartile based on the presence of CTLs, MKs, or both.

(G) 3D spatial visualization overlaying CTL and MK presence onto AML quartile information.

(H) 3D spatial visualization providing a higher magnification view of dense AML cluster 1, highlighting MK-rich and MK-devoid regions, with color shading indicating MK density.

AML, acute myeloid leukemia; CTLs, cytotoxic T lymphocytes; MKs, megakaryocytes.

CellPress
OPEN ACCESS

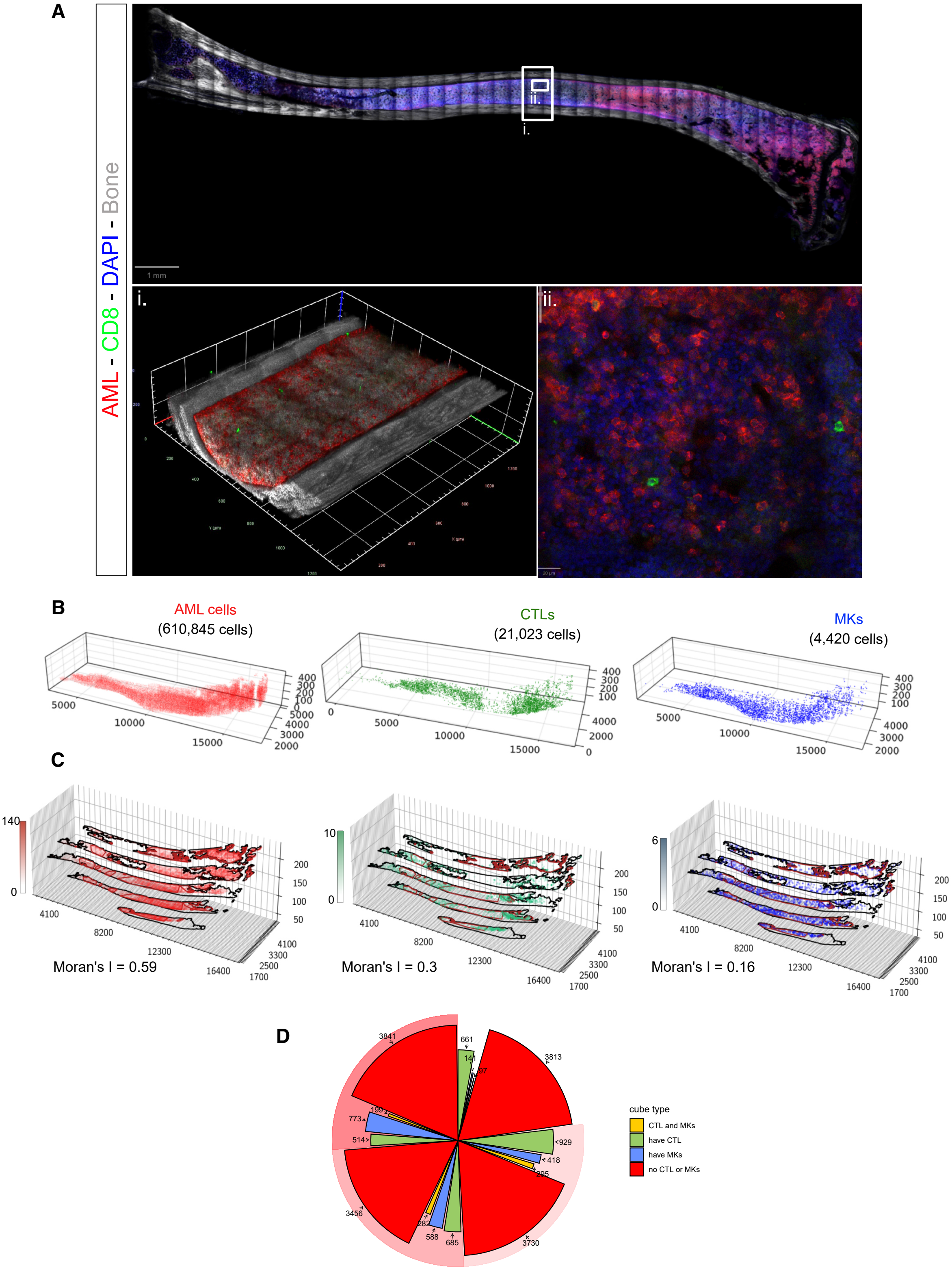

(legend on next page)

For 2D extraction, the YOLO neural network model was ideal because it proved to be highly effective, while minimizing time (approximately 30–120 min for images of similar size to the ones presented here) and computational power required. As images are provided to the model in their raw form, avoiding extensive image processing allows further time savings. Importantly, the model could be run efficiently not only on high-performance computing (HPC) clusters but also on cloud-based platforms, including free resources such as Google Colab. These characteristics provide the opportunity to analyze both larger samples, images acquired at higher resolutions, and images containing a higher number of cell types.

A known drawback of AI-based approaches is the need to generate manually annotated training data. Important for the classification of hematopoietic cells, the model was robust in identifying cells with irregular/variable morphology such as MKs, and reliable in cases where a cell type was very abundant, resulting in highly compact, adjacent cells, such as AML cells.[23,31,32] While we demonstrate the practical application of the model to three cell types, 2D neural networks such as YOLO are scalable and can be further trained, shared, and updated to include further cell types[23]; thus, our method to create 3D meaningful data is efficient regardless of the number of cell types. The effort required to train and optimize a neural network is, however, outweighed by the high returns achieved when applying the network to several images. The YOLO-based 2D framework is readily expandable with less extensive datasets through transfer learning.[31,32] Our pre-trained model easily identified other hematopoietic cell types within BM tissue, indicating it can be repurposed across tissues, analogous to the deposited gene signatures in transcriptomic studies. Furthermore, as 3D image analysis remains technically cumbersome, our strategy of aggregating 2D predictions into 3D structures offers a simple and effective alternative for quantitative 3D cellular mapping.

Previous studies have used image analysis to extract hematopoietic information from mouse BM and applied spatial statistical tests including Monte Carlo simulations, random dot analyses, and non-parametric tests (e.g., Mann-Whitney and Kolmogorov-Smirnov).[10–12,14] Building on these contributions, PACESS introduces a spatial statistical pipeline that improves the robustness and scalability of BM tissue spatial analyses. Although previous studies have successfully visualized cells of interest and their interactions within the BM microenvironment, the absence of statistical pipelines for spatial quantification, automated identification of regions of interest, and characterization of their neighboring relationships remains a limitation.[14–16,30] Addressing this, we incorporated Moran's I to enhance the spatial autocorrelation analysis. Additionally, our statistical approach enables direct quantification of regions of high cellular density and intercellular relationships within multiple cell types across the entire BM microenvironment, without relying on simulation-based estimates. By leveraging large-scale multicolor imaging datasets, our pipeline systematically captures spatial patterns that were previously difficult to quantify using traditional techniques.

While interest in leukemia and T cell interactions has been growing, stemming from immunotherapy developments, MKs have not been usually studied alongside the other two cell types. Our image analysis highlighted some unexpected spatial relationships between the three. Consistent with previous reports,[18,22] AML cells were identified forming several discrete clusters, with larger clusters in endosteal areas. The fact that no cell type returned a negative Moran's I value is consistent with the current view that hematopoietic cells are randomly distributed across BM space, with no overtly ordered topology. Interestingly, although several reports describe severe loss of all hematopoietic cell types as a consequence of AML growth,[18,22,33–35] our analysis highlighted that heavily infiltrated AML areas may not be homogeneous, as the BM area containing the largest AML cell cluster we observed could be split into two subregions, one devoid of both CTLs and MKs, and the other devoid of only CTLs.

This raises new questions about whether different healthy cell types are differentially displaced as AML progresses, and whether regions of maximal depletion reflect earlier sites of leukemic expansion.

In summary, the ultimate goal of quantitative 3D imaging is to informatively summarize, in a numerical form, the rich spatial and cellular information present within an image. This is particularly useful for settings where the principles regulating cell distribution are not clear, such as in BM tissue. PACESS provides a framework for the efficient generation, data extraction, and analysis of complex 3D BM tissue images. Importantly, this pipeline enables the analysis of images that would have proved too challenging to standard methodologies because of their size or complexity. The PACESS analytical workflow combines cell labeling and spatial statistical analysis to aid in the interpretation of spatial cellular data, which can provide a comprehensive insight into the relationships that exist within cell types in the BM. It promises to uncover the principles regulating the cellular organization of cells responsible for the lifelong production of blood cells and their deregulation when a hematological disease occurs.

Limitations of the study

PACESS requires a relatively long training phase, as model optimization and feature integration are computationally intensive.

Figure 5. Scaling the analysis to an entire bone section

(A) Tile scan of a single z position (z = 47 out of 92–200 GB) of a clarified 250-μm thick tibia section of an AML-infiltrated mouse. CTLs' cell membranes are labeled by CD8 immunostaining (green), AML cells express membrane-targeted tdTomato (red) and low level of intracellular GFP (green), nuclei are counterstained using DAPI (blue), and bone is imaged using SHG (gray). Inset (i) presents a 3D reconstruction of the identified bone region, and (ii) shows a high-magnification image.

(B) 3D reconstruction of AML cells (red), CTLs (green), and MKs (blue) within the bone marrow. The total number of identified cells for each population is indicated in black.

(C) Quantitative 3D visualization displays the spatial distribution of AML cells, CTLs, and MKs, with color scales indicating cell abundance. Moran's I values are reported for each cell type.

(D) Pie chart showing the classification of cubes within each AML density quartile based on the presence of CTLs, MKs, or both.

AML, acute myeloid leukemia; CTLs, cytotoxic T lymphocytes; MKs, megakaryocytes.

However, once trained, the model can be applied efficiently to new datasets in less time than the initial training. Another limitation is the need for HPC resources, as the training process demands substantial processing power and memory. While this may limit accessibility for some users, several free or academic HPC platforms are available that can support PACESS analyses. Future developments could focus on reducing computational requirements and improving accessibility to make PACESS more broadly usable.

RESOURCE AVAILABILITY

Lead contact

Requests for further information, resources, and reagents should be directed to and will be fulfilled by the lead contact, Cristina Lo Celso (c.lo-celso@imperial.ac.uk).

Materials availability

This study did not generate new unique reagents.

Data and code availability

- Microscopy images used in this study are available through the BioImage Archive (DOI https://doi.org/10.6019/S-BIAD1997)[36]: https://www.ebi.ac.uk/biostudies/bioimages/studies/S-BIAD1997
- All original code has been deposited at Zenodo: https://doi.org/10.5281/zenodo.17850017 and GitHub: https://github.com/ga402/PACESS.
- Any additional information required to reanalyze the data reported in this work paper is available from the lead contact upon request.

ACKNOWLEDGMENTS

We thank staff of the core facilities at Imperial College London (CBS facility, FILM) and the Crick Institute (BRF facility) for their valuable help. We thank Zeiss and Lorraine Berry for their support in the acquisition of the lightsheet images used in this study. We acknowledge the use of BioRender to generate Figure S1A. This work was supported by a Royal Society/Irish Royal Society International Exchange Program IEC\R1\180061 to C.L.C. and K.R.D.; CRUK Programme Foundation award C36195/A26770, Wellcome Trust Investigator Award 212304/Z/18/Z, and a Fondation ALCEA project grant to C.L.C.; and Science Foundation Ireland Project grant 18/CRT/6049 to K.R.D. G.A. was funded by a Wellcome trust 4i clinical PhD studentship (203928/Z/16/Z) and an NIHR Academic Clinical Lectureship. We also acknowledge the National Institute for Health Research (NIHR) Biomedical Research Centre based at Imperial College Healthcare NHS Trust and Imperial College London. The opinions, findings, conclusions, or recommendations expressed in this material are those of the authors and do not necessarily reflect the views of the NHS, the NIHR, the Department of Health, or Science Foundation Ireland.

AUTHOR CONTRIBUTIONS

G.A. and C.L.C. conceived the project and contributed to the experimental design with F.S.T.; G.A., F.S.T., and C.L.C. refined the bone immunofluorescence methods; G.A. and F.S.T. performed imaging experiments; C.M. contributed to imaging acquisition; G.A. labeled data, trained the neural network, and performed data extraction and classification; C.L., G.A., C.B., and K.R.D. devised the spatial statistical framework; and C.L. performed the statistical analysis. All authors contributed to the writing of the manuscript.

DECLARATION OF INTERESTS

The authors declare no competing interests.

STAR★METHODS

Detailed methods are provided in the online version of this paper and include the following:

- KEY RESOURCES TABLE
- EXPERIMENTAL MODEL AND STUDY PARTICIPANT DETAILS
  - Mice
  - AML experimental model
  - Intravital microscopy
- METHOD DETAILS
  - Immunofluorescence staining on thick bone sections
  - Image acquisition
  - Object detection and clustering
  - Spatial analysis and modeling
- QUANTIFICATION AND STATISTICAL ANALYSIS
  - Software
  - Statistical analyses

SUPPLEMENTAL INFORMATION

Supplemental information can be found online at https://doi.org/10.1016/j.crmeth.2026.101334.

Received: August 19, 2024
Revised: June 3, 2025
Accepted: January 27, 2026
Published: March 13, 2026

REFERENCES

1. Friston, K.J. (2011). Functional and effective connectivity: a review. Brain Connect. *1*, 13–36. https://doi.org/10.1089/brain.2011.0008.
2. Mesa, K.R., Kawaguchi, K., Cockburn, K., Gonzalez, D., Boucher, J., Xin, T., Klein, A.M., and Greco, V. (2018). Homeostatic Epidermal Stem Cell Self-Renewal Is Driven by Local Differentiation. Cell Stem Cell *23*, 677–686.e4. https://doi.org/10.1016/j.stem.2018.09.005.
3. Cheng, H., Zheng, Z., and Cheng, T. (2020). New paradigms on hematopoietic stem cell differentiation. Protein Cell *11*, 34–44. https://doi.org/10.1007/s13238-019-0633-0.
4. Laurenti, E., and Göttgens, B. (2018). From haematopoietic stem cells to complex differentiation landscapes. Nature *553*, 418–426. https://doi.org/10.1038/nature25022.
5. Kucinski, I., Campos, J., Barile, M., Severi, F., Bohin, N., Moreira, P.N., Allen, L., Lawson, H., Haltalli, M.L.R., Kinston, S.J., et al. (2024). A time- and single-cell-resolved model of murine bone marrow hematopoiesis. Cell Stem Cell *31*, 244–259.e10. https://doi.org/10.1016/j.stem.2023.12.001.
6. Zhang, J., Wu, Q., Johnson, C.B., Pham, G., Kinder, J.M., Olsson, A., Slaughter, A., May, M., Weinhaus, B., D'Alessandro, A., et al. (2021). In situ mapping identifies distinct vascular niches for myelopoiesis. Nature *590*, 457–462. https://doi.org/10.1038/s41586-021-03201-2.
7. Coutu, D.L., Kokkaliaris, K.D., Kunz, L., and Schroeder, T. (2018). Multicolor quantitative confocal imaging cytometry. Nat. Methods *15*, 39–46. https://doi.org/10.1038/nmeth.4503.
8. Nombela-Arrieta, C., Pivarnik, G., Winkel, B., Canty, K.J., Harley, B., Mahoney, J.E., Park, S.-Y., Lu, J., Protopopov, A., and Silberstein, L.E. (2013). Quantitative imaging of haematopoietic stem and progenitor cell localization and hypoxic status in the bone marrow microenvironment. Nat. Cell Biol. *15*, 533–543. https://doi.org/10.1038/ncb2730.
9. Khorshed, R.A., Hawkins, E.D., Duarte, D., Scott, M.K., Akinduro, O.A., Rashidi, N.M., Spitaler, M., and Lo Celso, C. (2015). Automated Identification and Localization of Hematopoietic Stem Cells in 3D Intravital Microscopy Data. Stem Cell Rep. *5*, 139–153. https://doi.org/10.1016/j.stemcr.2015.05.017.

10. Wu, Q., Zhang, J., Kumar, S., Shen, S., Kincaid, M., Johnson, C.B., Zhang, Y.S., Turcotte, R., Alt, C., Ito, K., et al. (2024). Resilient anatomy and local plasticity of naive and stress haematopoiesis. Nature *627*, 839–846. https://doi.org/10.1038/s41586-024-07186-6.

11. Kokkaliaris, K.D., Kunz, L., Cabezas-Wallscheid, N., Christodoulou, C., Renders, S., Camargo, F., Trumpp, A., Scadden, D.T., and Schroeder, T. (2020). Adult blood stem cell localization reflects the abundance of reported bone marrow niche cell types and their combinations. Blood *136*, 2296–2307. https://doi.org/10.1182/blood.2020006574.

12. Gomariz, A., Helbling, P.M., Isringhausen, S., Suessbier, U., Becker, A., Boss, A., Nagasawa, T., Paul, G., Goksel, O., Székely, G., et al. (2018). Quantitative spatial analysis of haematopoiesis-regulating stromal cells in the bone marrow microenvironment by 3D microscopy. Nat. Commun. *9*, 2532. https://doi.org/10.1038/s41467-018-04770-z.

13. Schienstock, D., Hor, J.L., Devi, S., and Mueller, S.N. (2025). Cecelia: a multifunctional image analysis toolbox for decoding spatial cellular interactions and behaviour. Nat. Commun. *16*, 1931. https://doi.org/10.1038/s41467-025-57193-y.

14. Gomariz, A., Portenier, T., Nombela-Arrieta, C., and Goksel, O. (2022). Probabilistic spatial analysis in quantitative microscopy with uncertainty-aware cell detection using deep Bayesian regression. Sci. Adv. *8*, eabi8295. https://doi.org/10.1126/sciadv.abi8295.

15. Gomariz, A., Portenier, T., Helbling, P.M., Isringhausen, S., Suessbier, U., Nombela-Arrieta, C., and Goksel, O. (2021). Modality attention and sampling enables deep learning with heterogeneous marker combinations in fluorescence microscopy. Nat. Mach. Intell. *3*, 799–811. https://doi.org/10.1038/s42256-021-00379-y.

16. Coutu, D.L., Kokkaliaris, K.D., Kunz, L., and Schroeder, T. (2017). Three-dimensional map of nonhematopoietic bone and bone-marrow cells and molecules. Nat. Biotechnol. *35*, 1202–1210. https://doi.org/10.1038/nbt.4006.

17. Akinduro, O., Weber, T.S., Ang, H., Haltalli, M.L.R., Ruivo, N., Duarte, D., Rashidi, N.M., Hawkins, E.D., Duffy, K.R., and Lo Celso, C. (2018). Proliferation dynamics of acute myeloid leukaemia and haematopoietic progenitors competing for bone marrow space. Nat. Commun. *9*, 519. https://doi.org/10.1038/s41467-017-02376-5.

18. Pirillo, C., Birch, F., Tissot, F.S., Anton, S.G., Haltalli, M., Tini, V., Kong, I., Piot, C., Partridge, B., Pospori, C., et al. (2022). Metalloproteinase inhibition reduces AML growth, prevents stem cell loss, and improves chemotherapy effectiveness. Blood Adv. *6*, 3126–3141. https://doi.org/10.1182/bloodadvances.2021004321.

19. Susaki, E.A., Tainaka, K., Perrin, D., Yukinaga, H., Kuno, A., and Ueda, H.R. (2015). Advanced CUBIC protocols for whole-brain and whole-body clearing and imaging. Nat. Protoc. *10*, 1709–1727. https://doi.org/10.1038/nprot.2015.085.

20. Li, W., Germain, R.N., and Gerner, M.Y. (2017). Multiplex, quantitative cellular analysis in large tissue volumes with clearing-enhanced 3D microscopy (Ce3D). Proc. Natl. Acad. Sci. USA *114*, E7321–E7330. https://doi.org/10.1073/pnas.1708981114.

21. Krivtsov, A.V., Twomey, D., Feng, Z., Stubbs, M.C., Wang, Y., Faber, J., Levine, J.E., Wang, J., Hahn, W.C., Gilliland, D.G., et al. (2006). Transformation from committed progenitor to leukaemia stem cell initiated by MLL-AF9. Nature *442*, 818–822. https://doi.org/10.1038/nature04980.

22. Duarte, D., Hawkins, E.D., Akinduro, O., Ang, H., De Filippo, K., Kong, I.Y., Haltalli, M., Ruivo, N., Straszkowski, L., Vervoort, S.J., et al. (2018). Inhibition of Endosteal Vascular Niche Remodeling Rescues Hematopoietic Stem Cell Loss in AML. Cell Stem Cell *22*, 64–77.e6. https://doi.org/10.1016/j.stem.2017.11.006.

23. Aloysius, N., and Geetha, M. (2017). A review on deep convolutional neural networks. 2017 International Conference on Communication and Signal Processing (ICCSP). https://doi.org/10.1109/ICCSP.2017.8286426.

24. Bodla, N., Singh, B., Chellappa, R., and Davis, L.S. (2017). Soft-NMS - Improving Object Detection with One Line of Code. Proceedings - 2017 IEEE International Conference on Computer Vision, ICCV 2017. https://doi.org/10.1109/ICCV.2017.593.

25. Buczkowski, M., Szymkowski, P., and Saeed, K. (2021). Segmentation of Microscope Erythrocyte Images by CNN-Enhanced Algorithms. Sensors *21*, 1720. https://doi.org/10.3390/s21051720.

26. Alsahafi, Y.S., Elshora, D.S., Mohamed, E.R., and Hosny, K.M. (2023). Multilevel Threshold Segmentation of Skin Lesions in Color Images Using Coronavirus Optimization Algorithm. Diagnostics *13*, 2958. https://doi.org/10.3390/diagnostics13182958.

27. Espindola, G.M., Camara, G., Reis, I.A., Bins, L.S., and Monteiro, A.M. (2006). Parameter selection for region-growing image segmentation algorithms using spatial autocorrelation. Int. J. Rem. Sens. *27*, 3035–3040. https://doi.org/10.1080/01431160600617194.

28. Schubert, E., Sander, J., Ester, M., Kriegel, H.P., and Xu, X. (2017). DBSCAN Revisited, Revisited: Why and How You Should (Still) Use DBSCAN. ACM Trans. Database Syst. *42*, 1–21. https://doi.org/10.1145/3068335.

29. Lehmann, E.L., and Romano, J.P. (2022). Testing Statistical Hypotheses (Springer International Publishing). https://doi.org/10.1007/978-3-030-70578-7.

30. Mertens, T.F., Liebheit, A.T., Ehl, J., Köhler, R., Rakhymzhan, A., Woehler, A., Katthän, L., Ebel, G., Liublin, W., Kasapi, A., et al. (2024). MarShie: a clearing protocol for 3D analysis of single cells throughout the bone marrow at subcellular resolution. Nat. Commun. *15*, 1764. https://doi.org/10.1038/s41467-024-45827-6.

31. Chanu, M.M., Lourembam, R., and Khelch, D. (2020). A Deep Learning Approach for Object Detection and Instance Segmentation using Mask RCNN. Journal of Advanced Research in Dynamic and Control Systems *12*, 95–104. https://doi.org/10.5373/JARDCS/V12SP3/20201242.

32. Lin, T.-Y., Dollár, P., Girshick, R., He, K., Hariharan, B., and Belongie, S. (2017). Feature Pyramid Networks for Object Detection. 2017 IEEE Conference on Computer Vision and Pattern Recognition (CVPR). https://doi.org/10.1109/CVPR.2017.106.

33. Miraki-Moud, F., Anjos-Afonso, F., Hodby, K.A., Griessinger, E., Rosignoli, G., Lillington, D., Jia, L., Davies, J.K., Cavenagh, J., Smith, M., et al. (2013). Acute myeloid leukemia does not deplete normal hematopoietic stem cells but induces cytopenias by impeding their differentiation. Proc. Natl. Acad. Sci. USA *110*, 13576–13581. https://doi.org/10.1073/pnas.1301891110.

34. Passaro, D., Di Tullio, A., Abarrategi, A., Rouault-Pierre, K., Foster, K., Ariza-McNaughton, L., Montaner, B., Chakravarty, P., Bhaw, L., Diana, G., et al. (2017). Increased Vascular Permeability in the Bone Marrow Microenvironment Contributes to Disease Progression and Drug Response in Acute Myeloid Leukemia. Cancer Cell *32*, 324–341.e6. https://doi.org/10.1016/j.ccell.2017.08.001.

35. Chen, L., Pronk, E., van Dijk, C., Bian, Y., Feyen, J., van Tienhoven, T., Yildirim, M., Pisterzi, P., de Jong, M.M.E., Bastidas, A., et al. (2023). A Single-Cell Taxonomy Predicts Inflammatory Niche Remodeling to Drive Tissue Failure and Outcome in Human AML. Blood Cancer Discov. *4*, 394–417. https://doi.org/10.1158/2643-3230.BCD-23-0043.

36. Hartley, M., Kleywegt, G.J., Patwardhan, A., Sarkans, U., Swedlow, J.R., and Brazma, A. (2022). The BioImage Archive - Building a Home for Life-Sciences Microscopy Data. J. Mol. Biol. *434*, 167505. https://doi.org/10.1016/j.jmb.2022.167505.

37. Muzumdar, M.D., Tasic, B., Miyamichi, K., Li, L., and Luo, L. (2007). A global double-fluorescent Cre reporter mouse. Genesis *45*, 593–605. https://doi.org/10.1002/dvg.20335.

38. Carrelha, J., Meng, Y., Kettyle, L.M., Luis, T.C., Norfo, R., Alcolea, V., Boukarabila, H., Grasso, F., Gambardella, A., Grover, A., et al. (2018). Hierarchically related lineage-restricted fates of multipotent haematopoietic stem cells. Nature *554*, 106–111. https://doi.org/10.1038/nature25455.

39. Hawkins, E.D., Duarte, D., Akinduro, O., Khorshed, R.A., Passaro, D., Nowicka, M., Straszkowski, L., Scott, M.K., Rothery, S., Ruivo, N., et al. (2016). T-cell acute leukaemia exhibits dynamic interactions with bone

CellPress
OPEN ACCESS

marrow microenvironments. Nature *538*, 518–522. https://doi.org/10.1038/nature19801.

40. Tissot, F.S., Gonzalez-Anton, S., and Lo Celso, C. (2024). Intravital Microscopy to Study the Effect of Matrix Metalloproteinase Inhibition on Acute Myeloid Leukemia Cell Migration in the Bone Marrow. Methods Mol. Biol. *2747*, 211–227. https://doi.org/10.1007/978-1-0716-3589-6_17.
41. Shaifee, M.J., Chywl, B., Li, F., and Wong, A. (2017). Fast YOLO: A Fast You Only Look Once System for Real-time Embedded Object Detection in Video. J. Comp. Vis. Imag. Sys. *3*. https://doi.org/10.15353/vsnl.v3i1.171.
42. Kharchenko, V., and Chyrka, I. (2018). Detection of Airplanes on the Ground Using YOLO Neural Network. 2018 IEEE 17th International Conference on Mathematical Methods in Electromagnetic Theory (MMET). https://doi.org/10.1109/MMET.2018.8460392.
43. Redmon, J., Divvala, S., Girshick, R., and Farhadi, A. (2016). You Only Look Once: Unified, Real-Time Object Detection. 2016 IEEE Conference on Computer Vision and Pattern Recognition (CVPR). https://doi.org/10.1109/CVPR.2016.91.
44. Weigert, M., Schmidt, U., Haase, R., Sugawara, K., and Myers, G. (2020). Star-convex Polyhedra for 3D Object Detection and Segmentation in Microscopy. IEEE Winter Conference on Applications of Computer Vision (WACV). https://doi.org/10.1109/WACV45572.2020.9093435.

STAR★METHODS

KEY RESOURCES TABLE

REAGENT or RESOURCE	SOURCE	IDENTIFIER
**Antibodies**		
CD8 antibody	ThermoFischer Scientific	MA1-145<br>Clone 2.43; RRID: AB_2536854
Laminin antibody	ThermoFischer Scientific	PA1-16730; RRID: AB_2133633
CD3	Abcam	ab5690; RRID: AB_305055
Donkey anti-Rabbit IgG (H + L) Highly Cross-Adsorbed Secondary Antibody, Alexa Fluor™ 594	ThermoFischer Scientific	A-21207; RRID: AB_141637
Anti-Rabbit IgG (H + L), highly cross-adsorbed, CF™ 633 antibody produced in donkey	Sigma	SAB4600132
**Bacterial and virus strains**		
pMSCV-MLL-AF9-IRESGFP	gift from Steve Lane (QIMR Barhofer, Brisbane)<br>Somervaille & Cleary. Cancer Cell 10, 257–268 (2006).	N/A
**Chemicals, peptides, and recombinant proteins**		
Dextran FITC	Sigma	FD2000S
Formaldehyde	ThermoFischer Scientific	28908
Low EEO agarose	Sigma	A0169
N,N,N′,N′-Tetrakis(2-Hydroxypropyl)ethylenediamine	Sigma	122262
Triton X-100	Sigma	X100
TBS	Fisher bioreagents	BP2471-1
DMSO	Sigma	D2665
Normal Donkey Serum	Sigma	D9663
DAPI	Invitrogen	D1306
N-Methlyacetamide	Sigma	M26305
1,4-Diazabicyclo[2.2.2]octane	Sigma	D27802
Histodenz	Sigma	D2158
Urea	ThermoFischer Scientific	29700
**Deposited data**		
Images available through the BioImage Archive[36]	This paper.	https://www.ebi.ac.uk/biostudies/bioimages/studies/S-BIAD1997<br>https://doi.org/10.6019/S-BIAD1997
**Experimental models: Organisms/strains**		
C57BL/6 mice	Charles River	N/A
mT/mG mice	Muzumdar et al.[37]	N/A
vWF-Tomato mice	Gift from Claus Nerlov and Sten Eirik Jacobsen (Oxford university) Carrelha et al.[38]	N/A
**Software and algorithms**		
YOLO-V5X model	This paper.	https://github.com/ultralytics/yolov5<br>https://doi.org/10.5281/zenodo.7347926
PACESS	https://github.com/ga402/PACESS	DOI:
Zen Blue	Zeiss	Carl Zeiss Zen 3.5 (Blue edition)

(Continued on next page)

Continued		
REAGENT or RESOURCE	SOURCE	IDENTIFIER
Zen Microscopy Software	Zeiss	Carl Zeiss Zen 3.1
Other		
Silicon isolator	ThermoFischer	P18175
Superfrost Plus^{TM} slides	Epredia	J1800AMNZ

EXPERIMENTAL MODEL AND STUDY PARTICIPANT DETAILS

Mice

All animal work was in accordance with the animal ethics committee (AWERB) at Imperial College London, UK and UK Home Office regulation (ASPA, 1986). All mice were bred and housed at Imperial College London or Sir Francis Crick Institute. C57BL/6 WT mice were purchased from Charles River (United Kingdom). vWF:tomato (von Villebrand Factor promoter driging tandem-tomato fluorescent protein expression) megakaryocyte reporter mice were a kind gift of Claus Nerlov and Sten Eirik Jacobsen (Oxford university). Male and female mice 6–16 weeks old were used. Sex did not evidently influence results in any of our analyses. Animals were housed in Tecniplast mouse greenline cages with appropriate bedding and enrichment. The temperature, humidity and light cycles were kept within the UK Home Office code of practice, with standard diet and water *ad libitum*, the temperature between 20 and 24°C, the room humidity at 45–65% and a 12-h light/12-h dark cycle with a 30-min dawn and dusk period to provide a gradual change.

AML experimental model

Murine AML cells were generated as described.[22] Briefly, granulocyte/monocyte progenitors (GMPs) were purified from mT/mG mice, transduced with pMSCV-MLL-AF9-GFP retroviruses as described[21] and transplanted into sub lethally irradiated mice (two doses of 3.3 Gy, at least 3 h apart). At 8+ weeks post-transplantation, recipient mice developed leukemia characterized by multi-organ infiltration. tdTomato positive cells were harvested from bone marrow and spleen and cells from each primary recipient were labeled as a separate batch and cryopreserved. Primary cells were thawed, suspended in PBS and 100,000 viable cells were injected i.v. into secondary, non-conditioned recipient mice.

Intravital microscopy

Live imaging of the calvarium bone marrow was performed as previously described.[39,40] Dextran FITC and tdTomato were excited using the 488nm and 561nm laser, respectively and detected with internal detectors.

METHOD DETAILS

Immunofluorescence staining on thick bone sections

Tibias, femurs and calvarium were harvested and fixed for 2h (unless otherwise specified in the text) in 4% formaldehyde at 4°C. Bones were decalcified for 10 to 15 days in 10% EDTA. Tibias and femurs were then embedded in 4% low EEO agarose (Sigma, A0169) and sectioned at 250 μm thickness using a Leica T1000 Vibratome. All the following steps were performed under agitation at room temperature. Sections or whole calvarium were incubated in 20% CUBIC-1 reagent (urea (25 wt % final concentration), Quadrol (25 wt % final concentration), Triton X-100 (15 wt % final concentration) in dH2O)[19] for 48 h and rinsed in TBS. At this stages, sections (or calvarium) can be stored at 4°C for up to ten days before immunostaining to ensure optimal staining quality; however, sections can be kept for a longer period of time for less sensitive immunostaining or when imaging reporters. To proceed to the immunostaining, unspecific antigen binding was avoided using blocking buffer solution (TBS 0.1% Triton, 10% DMSO, 5% normal donkey serum) overnight. The bones were then incubated with primary antibodies (CD8 clone 2.43 Invitrogen), diluted in blocking buffer for 48 h. After several consecutive incubations in washing buffer (TBS 0.1% Triton), bones were incubated with secondary antibodies for 48 h. Nuclei were counterstained using DAPI incubation over-night. Finally, the bones were mounted in the Ce3D clearing solution[20] (Methlyacetamide 40% (Sigma) diluted in TBS, 1.455g histodenz (sigma) per 1mL 40% Methlyacetamide, 4% DABCO (Sigma)) using silicon isolator (ThermoFischer P18175) on Superfrost Plus slides and imaged from 24 h later, once the tissue is cleared. Mounted samples can be stored at 4°C for several weeks without significant loss of signal.

Image acquisition

Image acquisition was performed using a Zeiss LSM 980 upright confocal microscope operated via Zen Blue 3.5 software and equipped with 5 Argon lasers (405, 488, 561,594 and 639nm), and an Insight (Newport Spectraphysics) 2-photon laser with two excitation lines of which one is fixed and one tunable (1045nm and 680-1300nm respectively), 6 non-descanned external detectors including 2 nose-piece detectors (GaASP). Images were acquired using a 20x, 1.0N.A., water immersion lens with 1.4mm working distance.

Lightsheet images (Figures S1G and S1H) were captured on a Zeiss Lightsheet 7 microscope operated via Zen Microsopy software version 3.1 with the 5x objective and dual PCO.edge sCMOS cameras (PCO.Imaging, Kelheim, Germany).

Object detection and clustering

The Imperial College High Performance Computing (HPC) cluster we used contains 8 CPUs and one P1000 GPU with 96 Gb of RAM and was accessed using a conventional laptop with 16GB RAM, Intel i9-9880H CPU with a Quadro T2000 Mobile GPU.

A YOLO-V5X model (https://github.com/ultralytics/yolov5) was used as the backbone of the 2D object-detection neural network. More details on this model, and on convolutional neural networks more generally, can be found in the literature.[23,41–43] In this section we provide only a brief overview of the model with a focus on the manner in which the algorithm identifies objects, and how it was adapted to generate 3D estimates. Image transformations, such rotation or artificial introduction of additional noise, are only used in the training and validation stages[44] to improve the neural network's performance when applied to unaltered experimental sample images.[43]

The YOLO algorithm works by placing a multitude of boxes within the space of a 2D image and then filtering these boxes based on probability estimates from the model. It is a fully connected neural network which divides a 2D image into $S \times S$ grid of cells into which B bounding boxes are detected. The model selects a range of box sizes for each class (in this case, biological cell type) using a k-means clustering algorithm run on the size of boxes observed within the training data, and uses it to label the test data. Each bounding box is defined by 5 parameters: the x, y central position, width (w), height (h) and a confidence score, C. This last value, C, is the confidence estimate over the presence, or absence, of an object being within the grid cell. This makes use of the intersection-over-union (IOU) between a predicted bounding box and a ground truth (manually annotated) bounding box. The greater the overlap between the two, the higher the IOU, and greater the confidence in the box. If any object is absent from the grid cell, the probability of the object (Pr(Object)) is set to 0. Otherwise it is 1. For the ith bounding box in j^{th} grid cell, the confidence score, C_{ij} is thus calculated as[43]:

$$C_{ij} = P\left(\text{Object}_{ij}\right) \times \text{IOU}$$

In addition to these five parameters a set of conditional class probabilities is calculated. Given K possible classes, this is the probability of the object belonging to any specific k^{th} class: $Pr(\text{Class}_k|\text{Object})$. A class-specific confidence score (CS_{kij}) is then calculated as a product of C_{ij} and the conditional class probability[43]:

$$CS_{ijk} = P\left(\text{Object}_{ij}\right) \times \text{IOU} \times \Pr\left(\text{Class}_{kij}\middle|\text{Object}_{ij}\right) = P\left(\text{Class}_{ijk}\right) \times \text{IOU}$$

The class-specific confidence scores and IOU results are used to select bounding boxes through non-maximum suppression (NMS).[24,43] YOLOv5 makes use of *soft*-NMS which is better adapted to overlapping objects.[24]

To aggregate the final set of 2D bounding boxes into 3D bounding 'cubes' we ordered the bounding boxes for each class by maximum diameter and mean fluorescence intensity (mFI). For each box within the set of boxes (B) within the kth class, starting from the largest and brightest boxes, a central x, y, z location is calculated, which we call q. We can also determine a maximum diameter for this box, d. From this point q the surrounding cluster of boxes in the z dimension which have a distance from q which is $<d/2$. We call this set of clustered bounding boxes B_c. Within this context $B_c \subset B$ but all the 2D bounding boxes within B_c are assumed to belong to a single cell (cube) surrounding an individual cell. Once identified, B_c is removed from the B. The process is repeated until every 2D box is allocated to a 3D cube. Of note, different cells have different brightness levels, however the absolute brightness of each cell does not affect this process, as the method adapts to variations in intensity. Each cluster of bounding boxes can have different brightness levels, ensuring that even weaker cells are correctly processed.

To apply this model, an $x \times y \times z \times c$ dimensional image, where $c = 3$ for RGB, was divided into a set of $416 \times 416 \times 1 \times 3$ (RGB) tiles. Test/validate/train subsets were selected from random sampling of this tile set. Manual annotation was performed to identify cells of interest within these selected images with a minimum of 500 cells annotated within each cell class. Once trained, the final object detection was performed on the full set of tiles.

Spatial analysis and modeling

Cells outside the bone marrow were excluded from the object detection dataset. The bone marrow was divided into $\theta(\mu m)^3$ cubes. The midpoint of the lowest plane in the cube (u,v,z) was used as the three-dimensional spatial coordinate, and the number of cells of each type in the cube was recorded. The data in each of these cubes was used as input to the analysis and model.

Moran's I index was used to measure spatial autocorrelation for each cell type.[27] The formula for Moran's I index is

$$I = \frac{n \sum \sum_{i \neq j} \varphi_{ij} (a_i - \bar{a})(a_j - \bar{a})}{\left(\sum_{i=1}^{n} (a_i - \bar{a})^2 \right) \left(\sum \sum_{i \neq j} \varphi_{ij} \right)}$$

where n is the total number of cubes, a_i is the number of cells in a particular type at the ith cube, a_j is the number of cells at the jth cube, $\bar{a}$ is the mean of the number of cells at each cube, and φ_{ij} is a spatial weight. The formula for φ_{ij} is

$$\varphi_{ij} = \begin{cases} 1 & \text{if } d_{ij} \leq \theta \\ 0 & \text{if } d_{ij} > \theta \end{cases}$$

where d_{ij} be the Euclidean distance between the centroids of cube i and cube j.

The density-based spatial clustering of applications with noise (DBSCAN) algorithm was used to identify clusters of cells.[28] For each cube (u_i,v_i,z_i), define $N_i = \{(u_j,v_j,z_j) | d((u_i,v_i,z_i),(u_j,v_j,z_j)) \leq \theta\}$ to be the set of all neighboring cubes that are distance θ μm or less away from the ith cube. The number of cells in N_i is recorded as $|N_i|$. When the faces of cubes are connected to each other, they are neighbors, so each central cube has six neighbors, for a total of 7 cubes, while cubes on a boundary have fewer neighbors. If the average number of cells in a cube (u_i,v_i,z_i) and its neighbors exceeds the third quartile of overall counts, then the ith cube and its neighbors are marked as high-density cubes. DBSCAN proceeds by identifying contiguous groups of high-density cubes as a cluster. The total number of clusters is the number of distinct contiguous high-density regions.

Permutation tests were used to detect changes in the number of cells in the cluster as well as the number of cells around the cluster.[29] The null hypothesis for the permutation test is that the mean number of cells in a cube is independent of whether the cube is in the cluster C_τ or is in the set of cubes adjacent to the cluster, C_τ^c. Let $|C_\tau|$ denote the number of cubes in the cluster and let $|C_\tau^c|$ record the number of cubes adjacent to the cluster. We define $A = \left(a_{\tau_1}, a_{\tau_2}, \cdots, a_{\tau_{|C_\tau|}}, a_{\tau_{|C_\tau|+1}}, a_{\tau_{|C_\tau|+2}}, \cdots, a_{\tau_{|C_\tau|+|C_\tau^c|}}\right)$ to be an ordered set of observations, where a_{τ_i} is the number of cells in the ith cube in the cluster for $i \leq |C_\tau|$ and the number of cells in a cube adjacent to the cluster for $|C_\tau| + 1 \leq i \leq |C_\tau| + |C_\tau^c|$. The following real-valued statistic is then used to test this null hypothesis:

$$M(A) = \left| \frac{\sum_{i=1}^{|C_\tau|} a_i}{|C_\tau|} - \frac{\sum_{j=|C_\tau|+1}^{|C_\tau|+|C_\tau^c|} a_j}{|C_\tau^c|} \right|$$

A permutation π is created, that reassigns labels to individual datum to obtain a reordered observation set

$$A_\pi = \left(a_{\pi(1)}, a_{\pi(2)}, \cdots, a_{\pi(|C_\tau|)}, a_{\pi(|C_\tau|+1)}, a_{\pi(|C_\tau|+2)}, \cdots, a_{\pi\left(|C_\tau|+|C_\tau^c|\right)}\right)$$

that also leads to a statistic $M(A_\pi)$. Under the null hypothesis, all permutations, Q, are equally likely. Thus, to execute the test, the empirical distribution of $M(A_\pi)_{\pi \in Q}$ is compared with $M(A)$. A Monte Carlo approximation with 500,000 random permutations was applied to estimate the two-tailed p-value, and the Holm-Bonferroni correction was performed.

The generalized linear model was used to predict the likelihood of the presence of cell types in a cluster versus not in an AML cluster. Each cube was changed into a dummy variable, as the response variable, based on whether it was in the cluster or not. The explanatory variables were determined by the presence or absence of the cell type. The generalized linear model was performed by 'glm' function in R (version 4.4.0).

QUANTIFICATION AND STATISTICAL ANALYSIS

Software

Zeiss Zen Blue version 3.1 software operated the LSM 980 to acquire images in confocal and two photon modality. Zeiss Zen version 3.1 software for Lightsheet was used to acquire images with dual-sided illumination. All PACESS source code was implemented in Python and is available on GitHub (see Data availability). Figures were generated primarily in Python 3.13 using geopandas (1.0.1), matplotlib (3.10.3), numpy (2.2.6), seaborn (0.13.2), pandas, shapely (2.1.1), scipy (1.15.2), scikit-learn (1.6.1), opencv (4.11), and pillow (11.2.1). 3D spatial visualizations additionally used geopandas, shapely, and scipy. Image-based figure panels incorporated opencv, pillow, and scikit-learn. The only exceptions are Figures 2D, 3A, and 4F, which were generated in R (version 4.4.3) using ggplot2 (3.5.2), scales (1.4.0), and ggrepel (0.9.6).

Statistical analyses

Statistical analyses were conducted using non-parametric methods where appropriate; for example, the p value in Figure 1D was computed using a Mann–Whitney U test. In Figure 1F, error bars denote the lower bound (Q1 − 1.5 × IQR) and upper bound (Q3 + 1.5 × IQR), with the central line indicating the median.

Cell Reports Methods, Volume *6*

Supplemental information

Practical AI-based cell extraction and spatial statistics for large 3D bone marrow tissue images

George Adams, Floriane S. Tissot, Chang Liu, Cera Mai, Chris Brunsdon, Ken R. Duffy, and Cristina Lo Celso

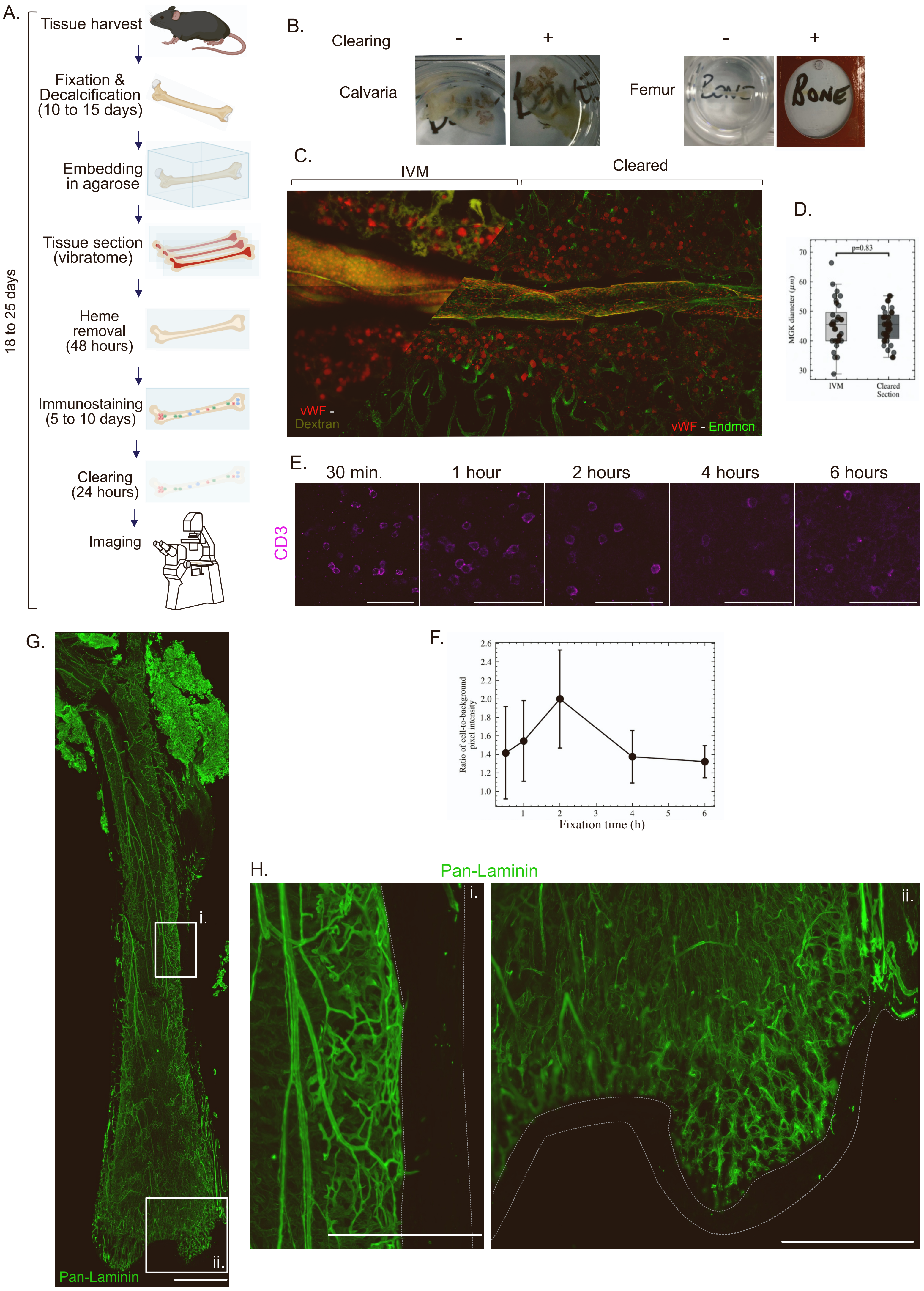
A.
Tissue harvest
Fixation &
Decalcification
(10 to 15 days)
Embedding
in agarose
Tissue section
(vibratome)
Heme
removal
(48 hours)
Immunostaining
(5 to 10 days)
Clearing
(24 hours)
Imaging
18 to 25 days
B.
Clearing
-
+
Calvaria
Femur
C.
IVM
Cleared
vWF - Dextran
vWF - Endmcn
D.
p=0.83
MGK diameter (μm)
IVM
Cleared Section
E.
30 min.
1 hour
2 hours
4 hours
6 hours
CD3
F.
Ratio of cell-to-background pixel intensity
Fixation time (h)
G.
i.
ii.
Pan-Laminin
H.
Pan-Laminin
i.
ii.

Figure S1

Figure S1: Bone marrow thick section preparation, related to Figure 1.

(A) Bone preparation workflow. Following harvest, the bones are cleaned, fixed for 2 hours and decalcified for 10-15 days. The bones are then embedded in 4% agarose and sectioned on a vibratome at a thickness of 250 µm. After heme removal (48h), sections are incubated with specific antibodies and cleared before imaging. **(B)** Comparison of the calvarium (left panels) and femur section (right panels) before and after clearing. **(C)** Effect of clearing protocol on bone marrow integrity. The same calvarium is imaged in vivo by intravital microscopy before undergoing the staining and clearing protocol and is re-imaged afterward. The left part of the image shows the IVM acquisition while the right side of the image display the post clearing acquisition. Blood vessels (green) are identified by FITC dextran intravenous injection in the IVM image and endomucin (Endmcn) immunostaining on the processed bone. MKs are identified thanks to vWF reporter transgene (red). **(D)** Quantification of MK size before (IVM) and after clearing protocol. **(E-F)** Effect of fixation time on immunostaining efficiency and background. **(E)** Representative images of each time point using the CD3 antibody (magenta). **(F)** The ratio cell to background is plotted. Scale bars = 50µm. **(G-H).** Blood vessels morphology after decalcification: lightsheet imaging of blood vessels identified via pan-Laminin immunostaining on a 250µm thick section prepared as described in (A). **(G)** Full femur image (scale bar = 1mm). Magnifications of bone lining marrow (i) and trabecular region (ii) are shown in **(H)** (scale bars = 500µm). The dashed lines indicate the bone determined by autofluorescence.

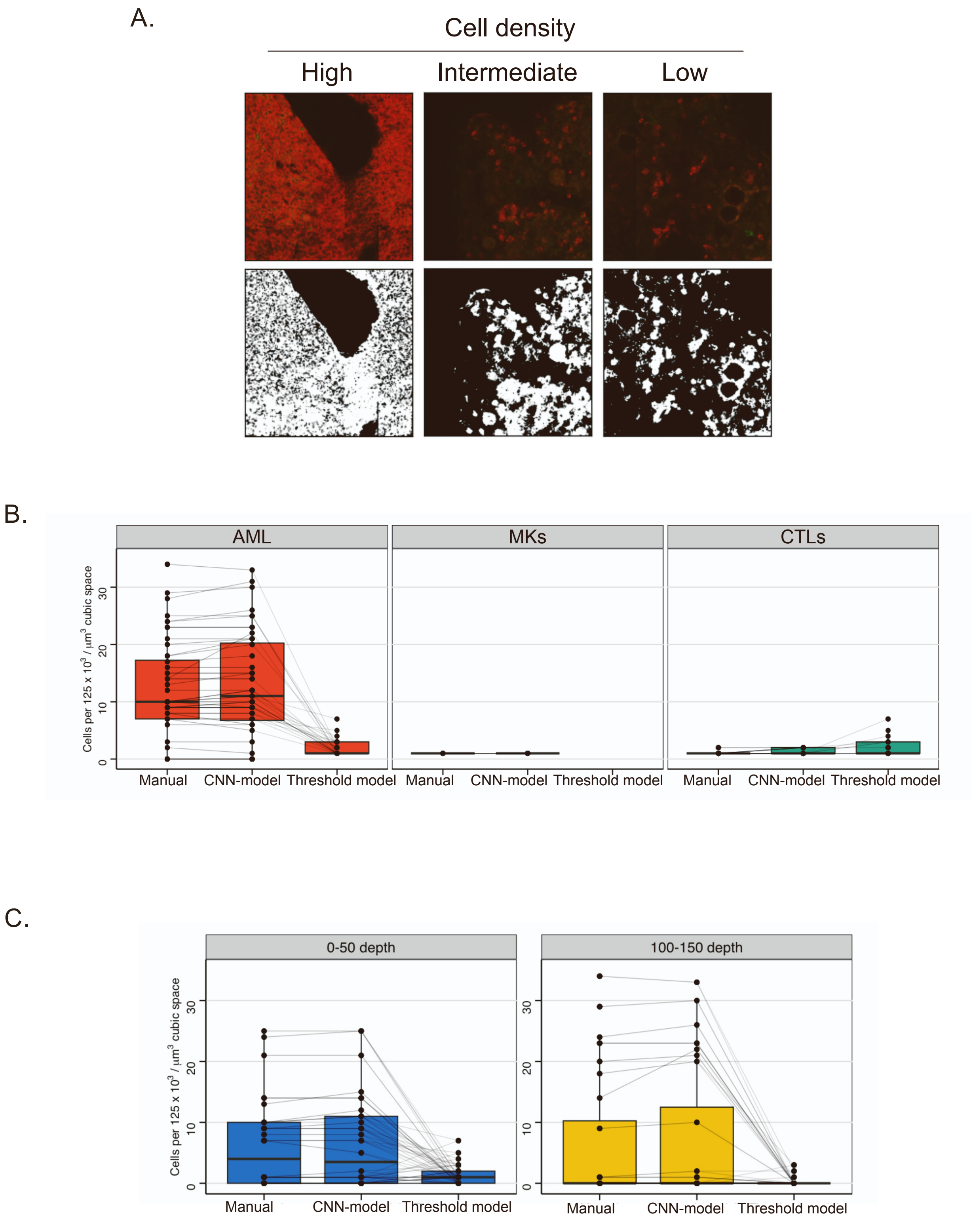

Figure S2: Comparison with threshold model of image analysis, related to Figure 1.
(A) Representative images showing the raw data (top) and the output (bottom) of the threshold model for AML cells. **(B)** Quantification of the number of cells identified using manual annotation, the neural network model and threshold model. **(C)** Comparison of each annotation model efficiency across different z-depths of the image.

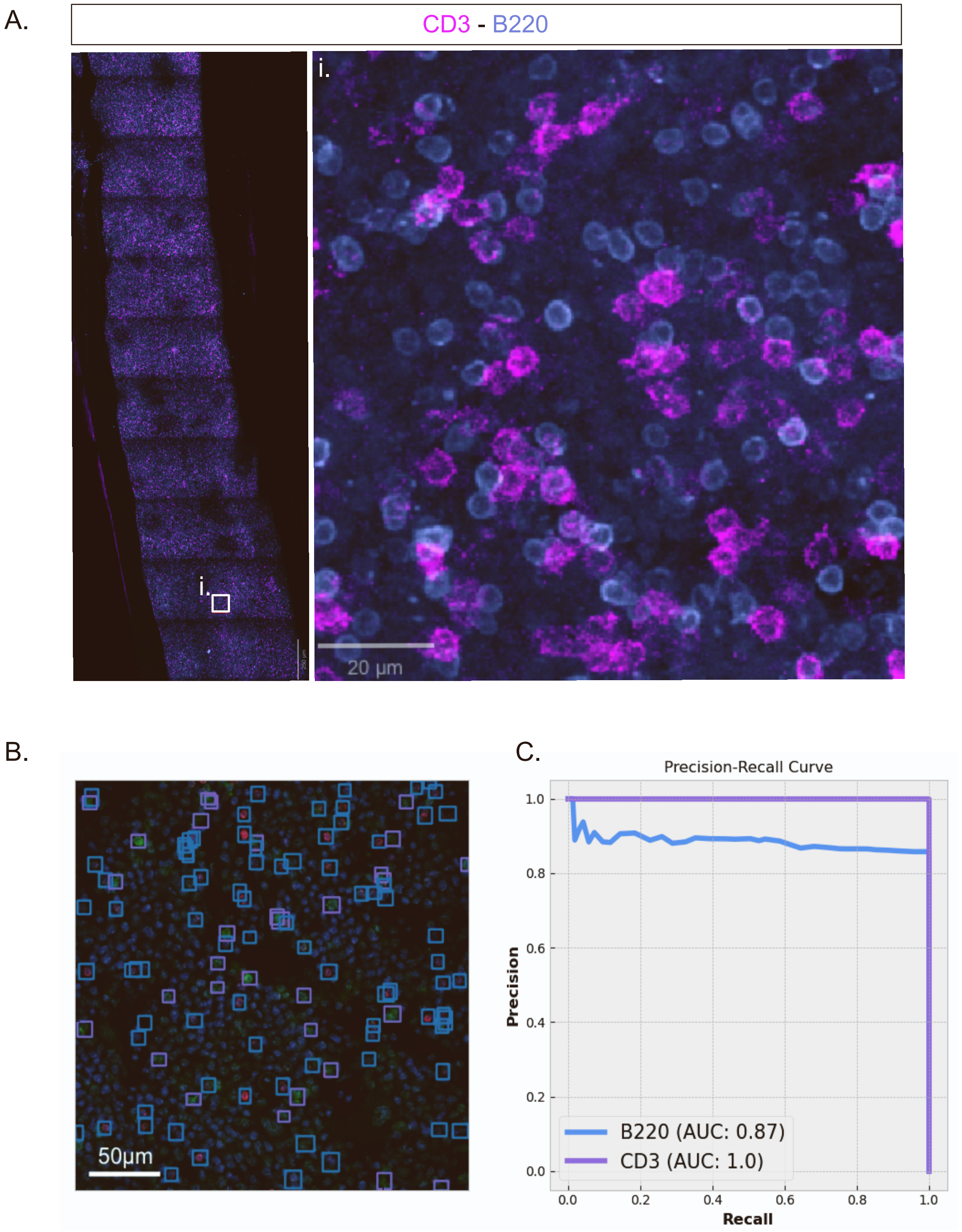

Figure S3: Neural network performance detecting bone marrow B and T cells, related to Figure 1. **(A)** Maximum intensity projection of five z-positions (z = 25 to 30 out of 39 total) of a 250 µm clarified mouse femur section. B ant T cells cell membranes are labelled by B220 and CD3 immunostaining respectively. Inset (i) shows a high-magnification image. **(B)** Representative example of B cell (blue boxes) and T cels (purple boxes) predictions generated by the 2D object detection. The model was applied without retraining from the version used in the main figures to detect MGKs, CTLs and AML cells **(C)** Precision-recall curve illustrating the performance of the 2D object detection model for each cell type.

A.

Moran's I

Cube size	AML cells	CTLs	MKs
45µm³	0.81	0.34	0.05
55µm³	0.82	0.45	0.09

B.

55µm³ cubes

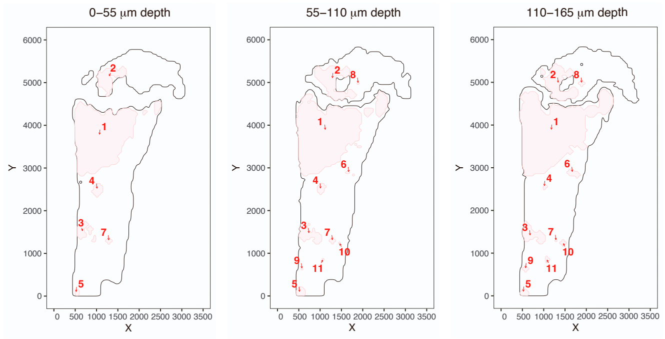

C.

45µm³ cubes	Coefficient	Exp(coef)	p-value
Only CTLs	-1.58	0.2051	< $2x10^{16}$
CTLs and MKs	-1.57	0.2085	< $2x10^{16}$
Only MKs	-0.06	0.939	0.34

55µm³ cubes	Coefficient	Exp(coef)	p-value
Only CTLs	-1.8	0.16	< $2x10^{16}$
CTLs and MKs	-1.8	0.16	< $2x10^{16}$
Only MKs	0.14	1.15	0.09

D.

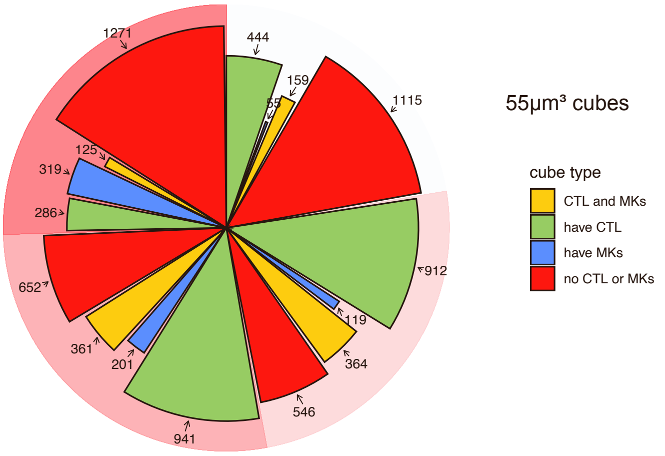

Figure S4: Effect of cube sizes on the spatial analysis, related to Figure 3.
(A) Table displaying the Moran's I values for various cube sizes. **(B)** 2D visualization of AML clusters identified by DBSCAN at a cube size set at 55µm3. The clusters are numbered in descending order based on the total AML cell count they contained. **(C)** Logistic regression results with the cube size of 45 µm3 (left) or 55 µm3 (right). **(D)** Pie chart showing the classification of 55µm3 cubes within each AML density quartile based on the presence of CTLs, MKs, or both.
